# Tuning the electronic structure of Ag-Pd alloys to enhance performance for alkaline oxygen reduction

**DOI:** 10.1038/s41467-021-20923-z

**Published:** 2021-01-27

**Authors:** José A. Zamora Zeledón, Michaela Burke Stevens, G. T. Kasun Kalhara Gunasooriya, Alessandro Gallo, Alan T. Landers, Melissa E. Kreider, Christopher Hahn, Jens K. Nørskov, Thomas F. Jaramillo

**Affiliations:** 1 Department of Chemical Engineering, Stanford University, 443 Via Ortega, Stanford, CA 94305 USA; 2 SUNCAT Center for Interface Science and Catalysis, SLAC National Accelerator Laboratory, 2575 Sand Hill Road, Menlo Park, CA 94025 USA; 3 Catalysis Theory Center, Department of Physics, Technical University of Denmark, 2800 Kongens, Lyngby, Denmark; 4 Department of Chemistry, Stanford University, 333 Campus Drive, Stanford, CA 94305 USA

**Keywords:** Catalysis, Electrocatalysis, Electrocatalysis, Fuel cells, Electronic structure

## Abstract

Alloying is a powerful tool that can improve the electrocatalytic performance and viability of diverse electrochemical renewable energy technologies. Herein, we enhance the activity of Pd-based electrocatalysts via Ag-Pd alloying while simultaneously lowering precious metal content in a broad-range compositional study focusing on highly comparable Ag-Pd thin films synthesized systematically via electron-beam physical vapor co-deposition. Cyclic voltammetry in 0.1 M KOH shows enhancements across a wide range of alloys; even slight alloying with Ag (e.g. Ag_0.1_Pd_0.9_) leads to intrinsic activity enhancements up to 5-fold at 0.9 V vs. RHE compared to pure Pd. Based on density functional theory and x-ray absorption, we hypothesize that these enhancements arise mainly from ligand effects that optimize adsorbate–metal binding energies with enhanced Ag-Pd hybridization. This work shows the versatility of coupled experimental-theoretical methods in designing materials with specific and tunable properties and aids the development of highly active electrocatalysts with decreased precious-metal content.

## Abstract (web summary)

Electrocatalyst development is key to improving the performance and viability of many electrochemical energy technologies. Here, the authors design Ag-Pd alloys with specifically tuned electronic structures to have enhanced oxygen reduction electrocatalysis and decreased precious metal content.

## Introduction

Large-scale deployment of fuel cells (FCs) and batteries could help lower anthropogenic CO_2_ emissions and create a more energy efficient society^1,2^. Hydrogen FCs (H_2_ FCs) are particularly interesting because H_2_ is an abundant commodity already produced at a large scale as an industrial chemical feedstock (~60 Mt y^−1^)^2^. Furthermore, H_2_ FCs can reduce up to ~50% of net CO_2_ emissions (relative to fossil fuel derived power) if hydrogen is produced by traditional methane reforming, and up to ~90% if hydrogen is produced by electrolysis with renewable electricity^1^. H_2_ FC vehicles are attractive for both light-duty and heavy-duty transportation as they can be refueled in minutes and provide sufficient energy density to be viable for a broad range of transportation modes including automobiles, trucks, buses, airplanes, and boats^3^. The large overpotential required for the oxygen reduction reaction (ORR) leads to low energy efficiency and is an important factor limiting the implementation of several electrochemical renewable energy technologies such as H_2_ FCs, direct methanol FCs, and metal-air batteries. Development of catalysts with improved activity, stability, and selectivity for the ORR will be necessary for the large-scale deployment of these technologies.

Based on guidelines from the U.S. Department of Energy, a large portion of H_2_ FC research and development has focused on lowering catalyst cost by decreasing the Pt content in proton exchange membrane FCs^3^. This has been done successfully by alloying and microstructuring^4–6^. However, owing to the thermodynamic instability of many first and second row transition metals in acidic conditions during the ORR, further alloy-based routes towards precious-metal elimination at low pH are limited^7–9^. In contrast, anion exchange membrane FCs (AEMFCs) offer a higher pH regime, enabling the stable use of less-expensive materials^9^. Currently, one of the most active known ORR catalysts in alkaline media is Pd^10–13^. Due to its high price, developing bimetallic Pd catalysts with decreased Pd content and/or higher intrinsic activity could make AEMFCs more economical. Ag shows promise as a secondary species because of its relatively low cost, high abundance, base stability, and high 4*e*^−^ selectivity^14–16^. Furthermore, previous experimental studies have demonstrated enhancement both in Ag-based bimetallics (e.g., Cu-Ag^17^, Co-Ag^18^, and Sn-Ag^19^) and Ag-Pd nano-configurations^20–23^, and physics-based modeling^24^ has predicted enhancement for the ORR based on *d*-band changes. Understanding the mechanism behind alloy-based enhancement could guide the discovery of structure-activity relationships for more complicated ternary or quaternary systems.

In this work, we use a thin film model system to study the compositional dependence of intrinsic activity for the ORR on Ag-Pd catalysts. Using electrochemical cycling, atomic force microscopy, x-ray diffraction, and x-ray spectroscopies we investigate activity, surface topography, crystal structure & solid miscibility, and composition & electronic structure, respectively. With our highly comparable alloyed catalysts, we demonstrate activities greater than that of pure Pd even after lowering Pd content by half. Using density functional theory together with these experimental results we probe the nature of the active site and rationalize the intrinsic activity trends seen for Ag-Pd electrocatalysts. By lowering the catalyst cost by ~50% via non-precious-metal alloying (Pd is currently ~100 times more expensive than Ag)^25^, without sacrificing overall performance, this work demonstrates an avenue for making H_2_ FCs and other electrochemical devices more economical.

## Results and discussion

### Alloying Ag and Pd as thin films

**Fig. 1:**
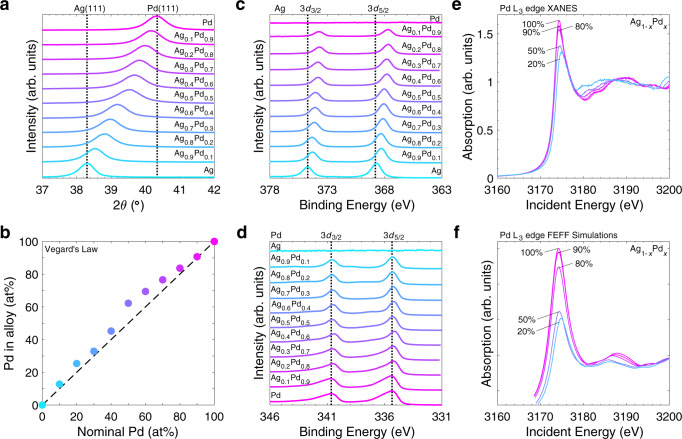
X-ray diffraction and x-ray spectroscopies of Ag-Pd thin films. **a** Grazing incidence x-ray diffractograms highlighting the (111) fcc peak as a function of Ag:Pd ratio in Ag_1-*x*_Pd_*x*_ films. Ag (38.30°) and Pd (40.35°) (111) peak locations are indicated with dotted lines. (See expanded 2*θ* range in Supplementary Fig. 1a, and symmetric-geometry diffractograms in Supplementary Fig. 2). **b** Vegard’s Law analysis of the (111) peak showing Pd content in the alloy versus nominal Pd composition. High resolution **c** Ag 3*d* and **d** P*d* 3*d* XPS spectra as a function of Ag:Pd ratio in Ag_1–*x*_Pd_*x*_ films (see survey spectra and XPS compositional analysis in Supplementary Figs. 3 and 4). **e** Normalized Pd L_3_ x-ray absorption near-edge region spectra (Pd L_3_-edge XANES) for select Ag_1–*x*_Pd_*x*_ compositions (*x* = 1, 0.9, 0.8, 0.5, 0.2), and **f** FEFF simulated Pd L_3_-edge XANES for select Ag_1-*x*_Pd_*x*_ compositions (*x* = 1, 0.9, 0.8, 0.5, 0.2). (See full XANES scan in Supplementary Fig. 5). All samples measured on standard glass slides for GI-XRD and on glassy carbon substrates for XPS and XANES. The colors refer to the nominal Pd loading, consistent with the text labels in the figure.

Ag-Pd alloys have been previously synthesized and are thermodynamically stable and miscible at all compositions^20–23,26–30^. Here, we synthesize a series of Ag_1-*x*_Pd_*x*_ (0 ≤ *x* ≤ 1) thin films (~70 nm thick) via electron-beam physical vapor deposition (PVD) and probe the crystal structure, extent of alloying, electronic structure, and topography of the as-prepared thin films with x-ray diffraction (XRD), x-ray photoelectron spectroscopy (XPS), x-ray absorption spectroscopy (XAS), and atomic force microscopy (AFM). Structurally, grazing incidence XRD (GI-XRD) (Fig. 1a, see full 2*θ* range for all Ag_1-*x*_Pd_*x*_ films in Supplementary Fig. 1a) of the pure Ag and pure Pd thin films shows strong peaks characteristic of a face-centered cubic (fcc) crystal structure with lattice parameters of *a* = 4.07 Å and *a* = 3.87 Å, respectively (see lattice data for all Ag_1–*x*_Pd_*x*_ films in Supplementary Table 1). Similar to their parent structures, the bimetallic Ag_1–*x*_Pd_*x*_ films have peaks corresponding to a fcc structure. Furthermore, at all compositions, the (111) peak ranges between the Ag(111) (38.30°) and Pd(111) (40.35°) peaks (Fig. 1a). The absence of two distinct (111) peaks in the bimetallic thin film diffractograms suggest that Ag and Pd are present as an alloy. Finally, the center of the (111) peak shifts linearly to higher diffraction angles as a function of increasing Pd composition. This linear shift is consistent with a decreasing lattice parameter as Pd is added to the Ag matrix. Using Vegard’s law (Fig. 1b), atomic alloy-compositions are seen to closely follow the nominal thin film composition (defined by the Ag and Pd masses as measured during evaporation), however slight deviations could suggest the presence of minor Pd-richer phases ~50% nominal Pd loading^31^. In short, XRD analysis highlights that electron-beam PVD produces highly miscible solid solution (alloy) phases of Ag_1-*x*_Pd_*x*_.

To probe the surface and clarify the role that composition plays in the electronic structure of the Ag_1–*x*_Pd_*x*_ alloys, we analyzed high resolution XPS (see survey spectra and XPS compositional analysis in Supplementary Figs. 3 and 4) and Pd L_3_-edge XAS. As seen in Fig. 1c, the binding energy of the Ag 3*d* peaks shifts negatively as a function of increasing Pd content, with a maximum shift of ~−1.0 eV between Ag and Ag_0.1_Pd_0.9_. This negative shift in the Ag 3*d* doublet, as a function of Pd content, has been seen previously for mixed Ag-Pd systems^21,26^ and has been attributed to increased charge mixing. Specifically, studies have shown that with increasing Pd content, the electron density in the Ag *d*-band increases, whereas the electron density in the *sp*-band decreases^21,28,32^. The Pd 3*d* (Fig. 1d) peak positions, however, vary only by a maximum of ~−0.3 eV. This small shift is in qualitative agreement with the majority of previously reported Pd 3*d* BE shifts in Ag-Pd alloys (mostly negative BE shifts);^21,26,28,32^ however, owing to instrument energy resolution it is difficult to draw trends as a function of Ag composition.

Although Pd XPS is complicated to interpret, the L_3_-edge XAS of Pd has been used effectively in the literature to elucidate the charge density within Ag-Pd alloys^23,32,33^. The small deviations from the single-particle-model for 4*d* metals makes the use of the L_3_-edge quantitatively meaningful^34^. Figure 1e, f (see Supplementary Fig. 5 for the full energy range plot) shows the experimental spectra and FEFF simulations of the Pd L_3_-edge for several Ag_1–*x*_Pd_*x*_ compositions. Looking at the x-ray absorption near-edge spectra (XANES), we see that the normalized white-line (Pd L_3_-edge peak) intensity decreases with increased Ag loading. This clear decrease in white-line intensity is indicative of a net shift in electron density from Ag to Pd producing a decrease of 4*d*-holes in Pd^32,33,35,36^. Moreover, the extended region features in Fig. 1e, f shift to lower energies with increased Ag loading in agreement with an expanding lattice owing to the addition of Ag (see Supplementary Fig. 5)^32,33,35,36^. The observed trends in the XANES of the Pd L_3_-edge (Fig. 1e) of Ag-Pd alloy thin films are well reproduced by FEFF simulations (Fig. 1f) of select Ag_1–*x*_Pd_*x*_ compositions (*x* = 1, 0.9, 0.8, 0.5, 0.2). Together, the Ag 3*d* XPS and Pd L_3_-edge XAS support a model, similar to that seen in previous Ag-Pd alloys^23,32,33^, in which the Ag and Pd *d*-bands become more filled as each metal dilutes the other. According to the *d*-band theory description of metal-adsorbate interactions, this change in *d*-band electron density has important catalytic implications that will be described further below.

Finally, AFM (Supplementary Fig. 6 and Supplementary Table 2), indicates that the surfaces of the as-prepared Ag_1–*x*_Pd_*x*_ thin films are very flat with roughness factors ≤1.02 at all compositions. This highly uniform surface morphology, regardless of composition, suggests that the geometric surface area is approximately equal to the exposed catalyst surface area for all films. Altogether, this in-depth physical characterization of the as-prepared Ag_1–*x*_Pd_*x*_ bimetallic thin films indicates that these materials have highly comparable, smooth surfaces. Furthermore, the unique electronic and physical properties as a function of Ag alloying with Pd, particularly the increase in *d*-band electron density and lattice expansion, motivates the investigation of their intrinsic catalytic activity for the ORR.

### Intrinsic ORR activity enhancement with Ag_1–*x*_Pd_*x*_ alloying

**Fig. 2:**
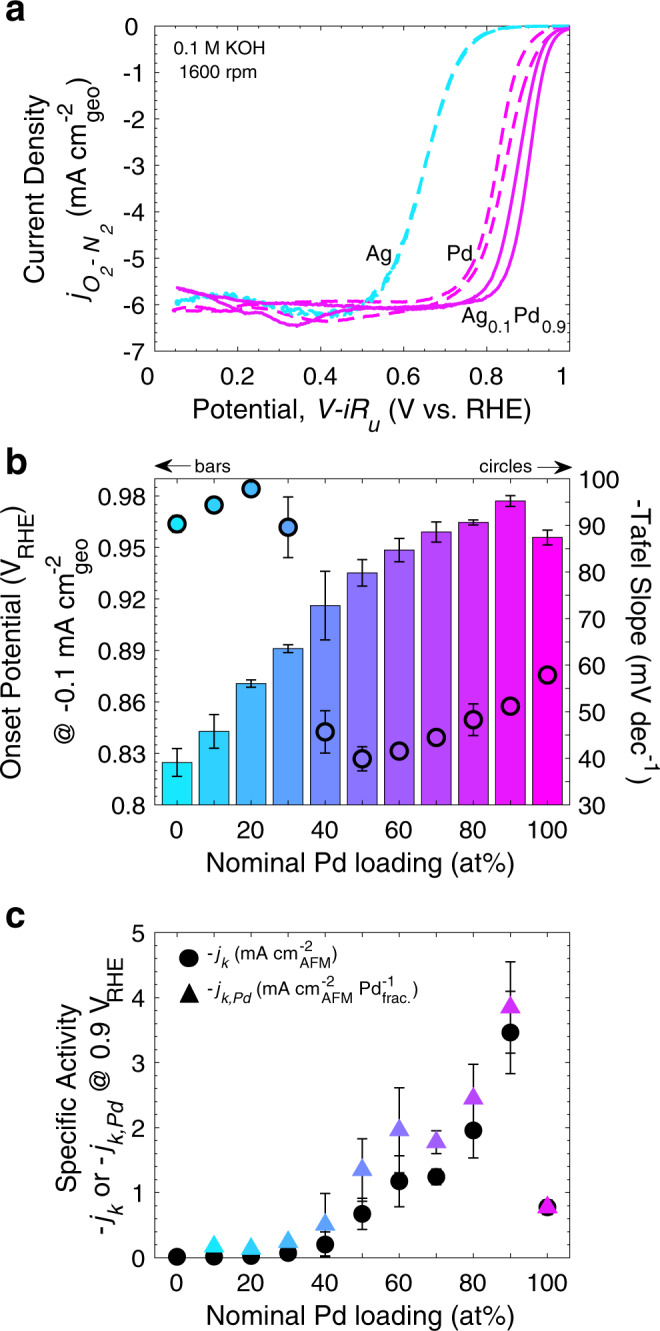
Electrochemical ORR evaluation of Ag-Pd thin film alloys. **a** Select ORR polarization curves for representative Ag, Pd, and Ag_0.1_Pd_0.9_ thin films. **b** Onset potential (bars) (potential at $- 0.1\,{\mathrm{mA}}\,{\mathrm{cm}}_{{\mathrm{geo}}}^{ - 2}$), Tafel slope (circles), and **c** specific activity (kinetic current density at 0.9 V vs. RHE normalized by surface roughness determined by AFM) on an absolute (black circles) and Pd fraction-normalized (color-mapped triangles) bases for all Ag_1-*x*_Pd_*x*_ compositions investigated. Measurements were taken at 1600 rpm in O_2_-saturated 0.1 M KOH, have non-ORR current subtracted (from CVs in N_2_-purged electrolyte), and are corrected for the uncompensated electrolyte series resistance. All data points are representative of the average value from the cathodic and anodic scan of the third CV cycle and error bars are the standard deviation from measurements with separate samples (see Supplementary Figs. 7, 11, 12 and Supplementary Table 3 for representative ORR CV curves, kinetic current densities at 0.9 and 0.8 V vs. RHE, representative Tafel plots, and tabulated data, respectively). The colors refer to the nominal Pd loading, consistent with the text labels in the figure.

The development of effective design principles for new materials requires characterizing and understanding the complexities and mechanisms behind activity enhancements. Using cyclic voltammetry (CV), we investigated the ORR activity as a function of alloy composition by cycling (20 mV s^−1^) various Ag_1–*x*_Pd_*x*_ thin films in 0.1 M KOH using a rotating disk electrode (RDE, 1600 rpm) (Fig. 2a and Supplementary Fig. 7). As seen in Fig. 2a and Supplementary Fig. 7, mass transport limited current densities are ~$- 6\,{\mathrm{mA}}\,{\mathrm{cm}}_{{\mathrm{geo}}}^{ - 2}$, indicating all compositions are selective to the four electron transfer pathway to ^–^OH ( < 0.5% H_2_O_2_ (*n*~3.98) above 0.3 V vs. the reversible hydrogen electrode (RHE) verified by rotating ring disk electrode and Koutecký–Levich (KL) selectivity measurements on Ag_0.1_Pd_0.9_, see Supplementary Figs. 8 and 9, respectively)^14–16,37^. The ORR polarization curves for pure Ag and pure Pd thin films are consistent with those of previous studies showing onset potentials (at $- 0.1\,{\mathrm{mA}}\,{\mathrm{cm}}_{{\mathrm{geo}}}^{ - 2}$) ~0.83 V and 0.96 V vs. RHE, respectively^14,17,38,39^. Furthermore, CV profiles in N_2_-purged electrolyte for pure Ag and Pd exhibit the redox features expected based on studies of single crystals and polycrystalline electrodes^17,21,40–44^. The bimetallic Ag_1–*x*_Pd_*x*_ thin films demonstrate a combination of these features (Supplementary Fig. 10). Figure 2a shows representative ORR voltammograms (with O_2_-saturation) for the Ag_1-*x*_Pd_*x*_ thin films, where *x* (%) = 0, 90, and 100. From this voltammetry we see that the highest performing composition, Ag_0.1_Pd_0.9_, has a very similar profile to the Pd control, indicating similar selectivity and reaction mechanism. However, this alloy outperforms both parent materials, on average, by a ~20 and ~55 mV lower overpotential, compared to Pd, at $- 0.1\,{\mathrm{mA}}\,{\mathrm{cm}}_{{\mathrm{geo}}}^{ - 2}$ (onset potential) and $- 3\,{\mathrm{mA}}\,{\mathrm{cm}}_{{\mathrm{geo}}}^{ - 2}$ (half-wave potential), respectively.

Probing the compositional dependence of this enhancement, we extracted the onset potential (Fig. 2b) and kinetic current density (Fig. 2c, calculated using the KL equation, see Supplementary Equation 1) from the polarization curves of the full Ag_1–*x*_Pd_*x*_ compositional range (Supplementary Fig. 7). In Fig. 2b we see that the onset potential for the ORR increases concomitantly with the Pd loading for all bimetallic films, with the most Pd-rich (80–90 at%) compositions onsetting, on average, ~10–20 mV before pure Pd. Moreover, after as much as a 60% decrease in Pd content, the onset potential remains above 0.9 V vs. RHE, indicative of a very high performing catalyst with significantly lowered precious-metal content. To look in depth at performance trends (Fig. 2c), the kinetic current was normalized based on the exposed catalyst surface area determined by AFM topography measurements (Supplementary Fig. 6 and Supplementary Table 2) to calculate a specific activity. Importantly, all activity trends remain the same when normalizing by electrochemical double-layer capacitance (potential cycling, Supplementary Fig. 13), geometric electrode area (Supplementary Fig. 11), and exposed catalyst surface area (AFM) (Fig. 2c), indicating that this specific activity trend is representative of the intrinsic activity trend. The specific activity was also normalized by Pd fraction to discern how the intrinsic activity of Pd atoms changes as a function of alloy composition.

As seen in Fig. 2c, there is a clear non-linear enhancement in specific activity at ≥50 at% nominal Pd loadings at 0.9 V vs. RHE. Specifically, films with 50–90 at% Pd have, on average, specific activities (on a Pd content and/or absolute bases) that are greater than both of their parent materials’ activity, with enhancements over pure Pd of 1.7–5.0× on a per Pd basis and 1.5–4.5× on a total specific activity basis. In addition, at 0.8 V vs. RHE (Supplementary Figs. 11 and 13a), a 1.4× specific activity enhancement is also present for Ag_0.6_Pd_0.4_ (not seen at 0.9 V vs. RHE due to a lower absolute current density). These activity enhancements suggest the presence of surface sites significantly more active for the ORR compared to pure Pd and Ag. Furthermore, when normalized by Pd content (Fig. 2c, triangles), the activity enhancements beyond the activity of pure Pd increase compared with those seen on a total specific activity basis (Fig. 2c, circles). This could suggest that when Pd is the majority species in the Ag_1–*x*_Pd_*x*_ alloyed thin films, Pd active sites are being enhanced.

The Tafel slope analysis of kinetic current densities further supports this Pd site enhancement hypothesis (Fig. 2b, circles). Specifically, there is a striking decrease in Tafel slope, concomitant with the appearance of specific activity enhancements, from ~90–100 mV dec^−1^ (Ag-like) to ~40–60 mV dec^−1^ (Pd-like) at Pd compositions of 40 at% or greater. Although the exact mechanistic change(s), leading to the observed differences in Tafel slope are difficult to pinpoint by Tafel analysis alone due to the multi-step nature of the ORR, this suggests intrinsic changes in the structure of the active site, the rate-limiting step, and/or the adsorbates are responsible for the observed enhanced ORR kinetics on the medium-to-high Pd content films^45,46^. The observed activity enhancements (specific activity at 0.9 V and 0.8 V vs. RHE) are only seen in the electrocatalysts with Tafel slopes similar to that of pure Pd. This could suggest a reaction mechanism similar to that of pure Pd, but in which Pd-based active sites are enhanced by Ag atoms within the lattice via ligand and/or strain effects.

In contrast to the medium-to-high Pd content catalysts, the films with ≤30 at% Pd show performance greater than that of pure Ag, but specific activities less than the linear combination of Ag and Pd activities at 0.8 V (or 0.9 V) vs. RHE. This suggests no overall synergistic effect between Ag and Pd at this low-Pd-content regime. Normalizing by Pd loading suggests that at this high-Ag-content compositional regime Pd atoms are playing an important role and are behaving as they do in their pure metal form (following an approximately linear activity-composition relationship). Moreover, Tafel slope analysis suggests that the reaction mechanism on the low-Pd-loading films is similar to that of pure Ag and supports the hypothesis that Ag is involved in the active site at this compositional regime. Despite no enhancement beyond the linear combination of Ag and Pd activities at this low-Pd-content regime, specific activity, and Tafel slope analyses suggest that both Ag and Pd are playing a role in increasing the overall performance above that of Ag. The composition-dependent role of Ag and Pd in modulating the ORR activity of Ag-Pd alloys will be described in the next section.

Post-reaction characterization of composition, structure, and surface smoothness suggests that the catalyst surface does not significantly change due to ORR testing on the timescale of our electrochemical measurements (see Supplementary Figs. 1, 4, 6, 14, and Supplementary Tables 1 and 2)^47^. This suggests the ex situ material properties can be meaningfully related to the activity trends, however, in operando physical characterization would be necessary to confirm the exact state of the catalyst surface during reaction. Most notably, the fact that surface smoothness did not change after ORR testing suggests that there was negligible surface restructuring (within AFM detection limits), an effect often associated with roughening^48^. To probe stability, we performed a chronoamperometry (CA) experiment at 0.8 V vs. RHE, for 2 hrs (Supplementary Fig. 14a), for our best-performing composition, Ag_0.1_Pd_0.9_, and observed a decrease in the ORR geometric current density of ~40%. We hypothesize that this decrease in performance, which we find to be recoverable, could be due to carbon uptake from corrosion of the graphite rod counter electrode, as well as possible Pd and/or Ag partial surface oxidation; the latter (oxidation) is in fact one of the main sources of decreased performance in similarly high performing Pt-based ORR catalysts^49^. ORR CVs pre- and post-CA testing (Supplementary Fig. 14b) support this hypothesis, as 100% of the initial (pre-CA) performance is recovered by cycling the working electrode down to ~−0.2 V vs. RHE to reductively clean off any accumulated carbon and/or surface oxide on the film’s surface. Additionally, using angle resolved (AR)-XPS (Supplementary Fig. 14c), to attain increased surface sensitivity, together with depth profiling (Ar^+^ sputtering, Supplementary Fig. 14d), we find that the composition of the film did not change significantly as a function of depth after stability testing^47,50^. Altogether, our stability testing and post-ORR in-depth characterization indicate that there is no irreversible intrinsic material or performance degradation on the timescale of our experiments, supporting the hypothesis that any trends in activity are related to intrinsic differences in material surface electronic structure rather than differences in catalyst morphology or any instabilities. Possible enhancement mechanisms for tuning the oxygen adsorbates binding energies correlated with these enhancements will be discussed in the next section.

Pure Pd catalysts have been noted among the most active materials for the ORR in alkaline media^12,13^. Here, we have shown that by alloying Ag with Pd we can both decrease the Pd content by 50% without activity loss and enhance the intrinsic activity of Pd five-fold, yielding high performing state-of-the-art ORR electrocatalysts. Supplementary Table 4 compares the specific activity (normalized by a physically representative exposed catalyst surface area) at 0.9 V vs. RHE of our best-performing thin films to representative state-of-the-art electrocatalysts for the ORR in 0.1 M KOH (or similar) using a RDE setup. Notably, our Ag_0.1_Pd_0.9_ alloy thin film electrocatalyst is among the most intrinsically active catalysts for the ORR in alkaline media yet reported when normalizing by exposed catalyst surface area. Although out of scope from this work, the high performance of the medium-to-high Pd content Ag-Pd alloys motivates future studies of this ideal catalyst system within FC systems.

### Electronic effects on ORR activity from alloying Ag and Pd

**Fig. 3:**
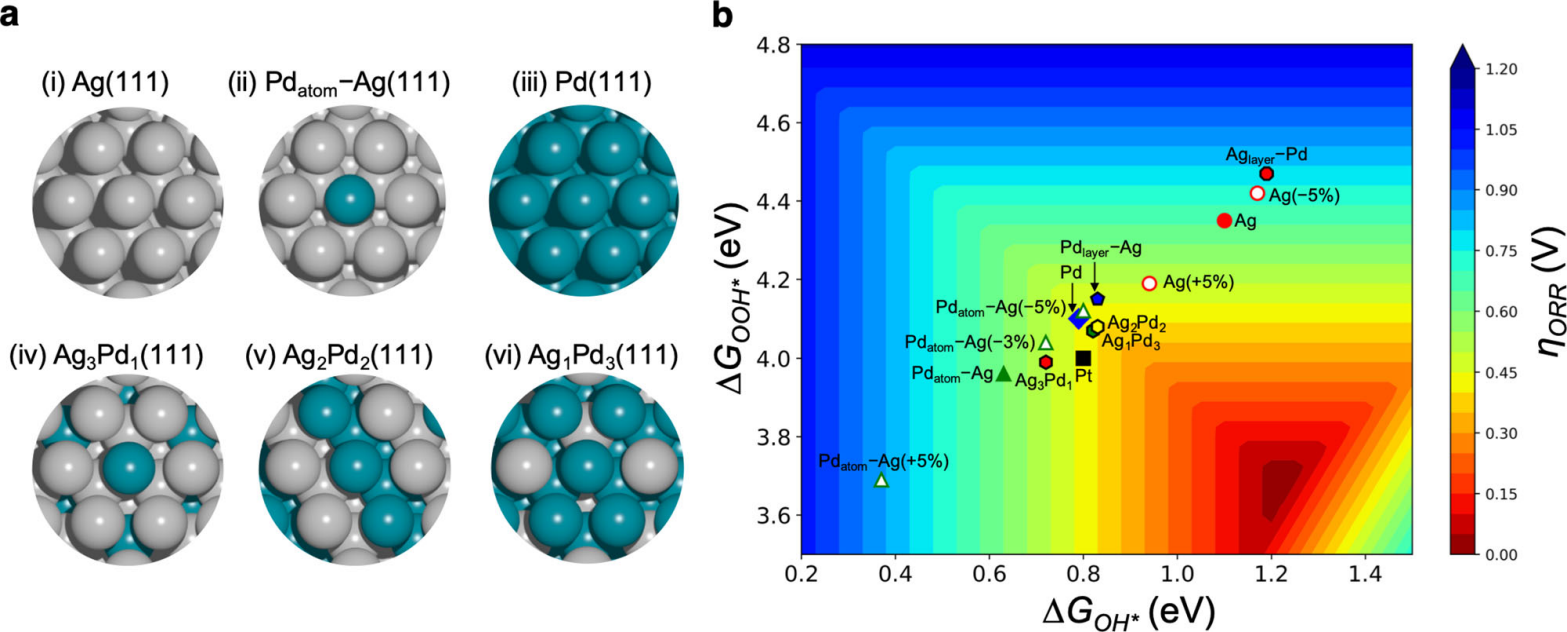
DFT-calculated ORR activity on Ag-Pd active site motifs. **a** Ag-Pd active site models investigated by DFT for their effect on ORR activity: (i) Ag, (ii) Pd_atom_–Ag, (iii) Pd, (iv) Ag_3_Pd_1_, (v) Ag_2_Pd_2_, and (vi) Ag_1_Pd_3_. All the considered surfaces are (111) facets. The identified most active sites are: (i) Ag_top_, (ii) Ag–Pd, (iii) Pd–Pd, (iv) Ag–Pd, (v) Pd–Pd, and (vi) Pd–Pd. **b** Oxygen reduction reaction (ORR) activity map showing overpotentials (vs. the computational hydrogen electrode) for various Ag-Pd structures calculated by DFT. *η* = 1.23 V − *U*_*L*_. (The ORR *U*_*L*_ is defined as the highest potential at which all the reaction steps become downhill in energy. Lower *η* or higher *U*_*L*_ indicate improved theoretical ORR activity). See Supplementary Figs. 15–17 and Supplementary Tables 5 and 6 for more details about strain effects, projected density of states, and the tabulated data.

Based on the *d*-band model, which describes metal-adsorbate orbital hybridization, it is largely understood that there is an optimal *OH/*OOH binding energy for ideal ORR catalysis^51–54^. Ag binds oxygen adsorbates too weakly, while Pd binds them too strongly^54,55^. Hence, the alloying of Ag and Pd might lead to more optimal catalysts, with stronger Ag- or weaker Pd-adsorbate binding^24,51–55^. Using density functional theory (DFT) we can investigate the theoretical activity for the ORR as a function of Ag-Pd structure and composition. Therefore, a complementary theoretical study was undertaken to gain a fundamental understanding of the nature of the active sites and trends in activity to rationalize the experimental observations, namely the physical and electronic properties and the enhanced ORR activity on Ag-Pd alloys compared with their pure components. First, we identify the composition of the most active sites and then determine the role of strain (tensile or compressive) and ligand (hybridization) effects, both of which can arise from alloying, in modulating the electronic structure within these active sites. We consider a variety of Ag-Pd (111) configurations representative of the synthesized materials (Fig. 3a), as well as all possible active sites, to deconvolute strain from ligand effects. We evaluate activity based on the calculated thermodynamic limiting potential for the ORR (*U*_*L*_)^56^, which is dependent on the surface binding energies of reacting oxygen adsorbates (Fig. 3b).

In Ag-Pd alloys, the absolute strain, seen by changes in bond lengths, on each atom (Ag or Pd) changes as a function of the Ag:Pd ratio. This is because, as seen by XRD (Fig. 1a), Pd has a smaller lattice constant than Ag. As a function of this difference in lattice parameter, Pd and Ag atoms will be under increased tensile or compressive strain that strengthens or weakens the adsorption energy of reaction intermediates as a function of Pd content, respectively. Therefore, strain would only lower the activity of pure Ag- or Pd-like active sites (we show a quantitative example of this in Supplementary Fig. 15a). Based on this traditional understanding of strain effects, we would expect no enhancement in activity for the Ag-Pd system. However, there are additional electronic effects to consider for active sites that share electrons locally. To model the activity of Ag-Pd alloys resembling the experimental Ag-Pd thin films, the ORR *U*_*L*_ was calculated for the Ag_3_Pd_1_(111), Ag_2_Pd_2_(111), and Ag_1_Pd_3_(111) structures (structures (iv)–(vi) in Fig. 3a). Our calculations suggest that the most active (lowest energy) sites for the Ag_2_Pd_2_(111) and Ag_1_Pd_3_(111) surfaces are Pd–Pd bridge sites, whereas the active site for Ag_3_Pd_1_(111) is the Pd–Ag bridge site. The theoretical activity results for these alloy structures, as well as for Pt(111), Pd(111), and Ag(111) are shown in Fig. 3b. The limiting potentials for Pt(111) and Pd(111) are 0.80 V and 0.79 V, respectively. The limiting potentials for Ag_2_Pd_2_(111) and Ag_1_Pd_3_(111) are 0.83 V and 0.82 V, respectively, and are slightly higher than for pure Pd(111) indicating enhancement of the Pd–Pd bridge sites in medium-to-high Pd content Ag-Pd alloys. The *d*-projected density of states analysis (see Supplementary Fig. 16) for the structures in Fig. 3a illustrates that the slightly lower *d*-band edge relative to the Fermi level observed for Ag_1_Pd_3_(111) and Ag_2_Pd_2_(111) compared with Pd(111) results in a weaker oxygen adsorption on Pd atoms and consequently an increase in activity. As illustrated by the Pd L_3_-edge XANES measurements (Fig. 1e) discussed earlier and the calculated Pd effective Bader charge (Supplementary Table 6), this change in Pd *d*-band arises from the increased filling of Pd 4*d*-holes upon charge hybridization between Ag and Pd. Therefore, we hypothesize activity enhancements on medium-to-high Pd content electrocatalysts are likely due to ligand effects, specifically optimal charge redistribution between Ag and Pd. Furthermore, the presence of an increased number of active Pd−Pd bridge sites may explain why the experimental activity increases with Pd content despite Ag_2_Pd_2_(111) being slightly more theoretically active than Ag_1_Pd_3_(111).

Considering the Ag-rich compositional regime, the calculated limiting potential for the ORR on Ag_3_Pd_1_(111) (0.72 V) improved relative to Ag(111) but does not reach the enhancement observed in the Ag_2_Pd_2_(111) and Ag_1_Pd_3_(111) surfaces. To consider strain effects on the predicted mixed-metal active site for structures with <50 at% Pd, we calculated the *U*_*L*_ of the Ag−Pd bridge site in a Ag(111) surface with a trapped single Pd atom (Pd_atom_−Ag) (structure (ii) in Fig. 3a). The calculated *U*_*L*_ of Pd_atom_−Ag is 0.63 V, indicating a slight increase in activity compared to pure Ag(111) with a *U*_*L*_ of 0.61 V. For this system, the ORR *U*_*L*_ increases with compressive strain (lattice contraction weakens adsorbate binding), indicating that the Pd_atom_−Ag site lies on the strong binding side of the ORR volcano, as shown in Fig. 3b. With a compressive strain of 5%, Pd_atom_−Ag (−5%) shows a calculated *U*_*L*_ of 0.80 V (see Supplementary Figs. 15b and 17a); tensile strain (lattice expansion), on the other hand, leads to a significant decrease in *U*_*L*_. Ag_3_Pd_1_(111), specifically, has an active site similar to Pd_atom_−Ag(−2%) (*U*_*L*_ of 0.66 V), and compressive strain (relative to Ag(111)) arising from the addition of Pd could partly lead to a higher *U*_*L*_, but strain alone does not explain the higher *U*_*L*_ for Ag_3_Pd_1_(111) compared with Pd_atom_−Ag(−2%). Therefore, ligand effects are likely also playing a role in increasing the *U*_*L*_ of Ag_3_Pd_1_(111). Consequently, we conclude that the increased *U*_*L*_ for the Ag_3_Pd_1_(111) structure is due to the intrinsic electronic interactions between Ag and Pd at such Ag-rich compositions, as well as strain on the Ag–Pd site. However, as suggested by the specific activity of the low-Pd-loading thin films not reaching the linear combination of their parent materials’ activity, the charge hybridization of Ag and Pd, as well as compressive strain, have a suboptimal effect at this compositional regime. Altogether, the DFT calculations on Ag-Pd structures of different composition agree well with the activity enhancement observed in Ag-Pd thin films with medium-to-high Pd-loading, and the lack of synergistic enhancement (but still higher activity than pure Ag) at lower Pd loadings.

Overall, DFT results on mixed-metal structures along with the experimentally observed specific activities for the ORR suggest that the most likely mechanism for activity enhancement is the electronic structure tuning of Pd–Pd active sites induced by the surrounding bonding environment (ligand effects) in medium-to-high Pd content Ag-Pd alloys. For the thin films with low-Pd loading, there is still an increase in limiting potential and onset potential for the ORR relative to pure Ag, but the specific activity is less than the expected linear combination of Ag and Pd activities. Therefore, we can conclude that the Ag-Pd electronic interactions at this low-Pd-loading regime do not enhance ORR activity. Correlating the DFT, XPS, and Pd L_3_-edge XANES results for the Ag-Pd alloy thin films, the observed activity enhancements for films with ≥40 at% Pd are attributed to electronically enhanced active Pd–Pd sites, which we hypothesize have optimized Pd and Ag *d*-band filling and center.

In conclusion, Ag_1-*x*_Pd_*x*_ thin films, with 0 ≤ *x* ≤ 1, were synthesized by electron-beam PVD. XRD and XPS measurements indicate that the metals alloyed, and the films were created with the intended nominal composition, which remained approximately constant after electrochemical testing. In alkaline conditions, the ORR activity of the thin films with ≥ 40 at% Pd loading surpassed the linear combination of Ag and Pd activities. In addition to very high activity, Ag_0.1_Pd_0.9_ exhibited the most enhanced ORR intrinsic specific activity, on both an exposed catalyst surface area and Pd content bases. Overall enhancements over pure Pd were found down to 50 at% Pd loading, with the intrinsic activity of these films (50–100 at% Pd) among the highest recorded. Based on experimental results and DFT calculations, the observed activity enhancements are mainly attributed to electronically enhanced Pd–Pd active sites leading to improved oxygen binding energies. In this study, we leverage the interplay between alloying, electronic structure, and catalytic activity and provide a platform to tune the local active site environment of materials to make more economical state-of-the-art ORR catalysts.

## Methods

### Thin film synthesis

Bimetallic thin films were synthesized via electron-beam physical vapor co-deposition (Ag with 99.99% purity and Pd with 99.95% purity targets from Kurt J. Lesker) using a custom made PVD system (Technical Engineering Services) equipped with two electron beams, a thermal source, and a rotating substrate stage. Each source is equipped with an independently calibrated quartz crystal microbalance to allow careful deposition rate and composition control throughout the thin films. Prior to deposition, pre-polished 5 mm diameter glassy carbon disks (HTW Hochtemperatur-Werkstoffe GmbH) were cleaned by sequential sonication for 30 min in soapy water, acetone, ethanol, and isopropanol. Subsequently, they were rinsed in pure Millipore water (*R* = 18 MΩ cm), and affixed to a 10 cm diameter Si(100) wafer using double-sided Kapton tape. We made 70 nm thick Ag-Pd thin films with individual deposition rates (≤0.11 nm/s individually) set to achieve the desired target bimetallic composition ranging from pure Ag to pure Pd. The electron-beam(s) sweep pattern was an up and down (vertical) sweep motion of an ellipse-shaped beam (elongated in the horizontal axis). We found this sweep pattern to produce more uniform thin films with accurate bulk and near-surface composition. A sticking layer was not used due to adhesion problems, observed in early syntheses, between Ag-Pd thin films and investigated sticking layers (tested 2 nm, 10 nm, 20 nm of Ti or Cr).

### Material physical characterization

XPS was performed using a PHI III Versaprobe equipped with Al Kα radiation (1486 eV). Survey spectra were collected using a 224 eV pass energy, and high-resolution spectra were obtained using a 55 eV pass energy. Spectra were collected employing the instrument’s neutralizer and the Ar^+^ gun in neutralizing mode. The x-ray source was perpendicular to the sample and the sample-to-detector angle was 45° (default). A 200 μm × 200 μm spot was used, and the spot location was set to approximately the center of the samples. The stage height was auto-aligned for each sample-to maximize signal intensity. Compositional quantification of XPS spectra was done using CasaXPS software. Tougaard background corrections were used to calculate the area under the Pd and Ag 3*d* peaks (manually selected energy range) to subsequently calculate their near-surface atomic composition (with sensitivity factors: S_Ag, 3*d*_ = 18.04 and S_Pd, 3*d*_ = 16.04). XPS spectra were aligned by setting the C 1*s* peak to 284.8 eV. XAS was performed at beamline 14-3 at the Stanford Synchrotron Radiation Light Source at SLAC National Accelerator Laboratory in a He-saturated chamber. A small glitch in the data is seen at 3202 eV coming from trace Ar in the He. A four element Vortex detector was used to collect fluorescence from the samples. All XAS data were processed and edge jump normalized using Athena^57^ software. We obtained 5 μm × 5 μm size non-contact AFM images using a Park Systems XE-70 instrument equipped with a premounted Park Systems NSC15/Al BS tip and calculated topographical information using Gwyddion^58^ software. XRD measurements were collected on a PANalytical Materials Research diffractometer equipped with a Cu Kα (*λ* = 0.154 nm) x-ray source. Using a 20 mm beam mask, and a 1/2 mm divergence slit, we collected grazing incidence ($\omega = 1^\circ$) measurements and symmetric scans (see Supplementary Figs. 1 and 2) on Ag-Pd thin films grown on standard glass slides to increase the area of material under illumination and eliminate background interferences from glassy carbon substrates. After electrochemical testing, grazing incidence ($\omega = 4.4^\circ$) diffractograms on Ag-Pd thin films grown on glassy carbon substrates were collected employing a 5 mm beam mask, and a 1/8 mm divergence slit. Owing to the low signal to noise ratio in the diffractograms for the samples on glassy carbon substrates, OriginPro was used to fit a Lorentzian distribution profile to these data to quantitatively determine the (111) peak position. Post-electrochemical (removed from electrolyte at the open circuit potential and rinsed with Millipore water (*R* = 18 MΩ cm)) physical characterization (XRD, XPS, AFM) was performed on all thin films within 1 month of activity testing (stored in air).

### Electrochemical/activity characterization

Electrochemical characterization was performed by RDE (1600 rpm) testing using a Pine Research Instruments rotator setup and a Biologic VMP-300 potentiostat. We used a three-electrode cell, and employed a graphite rod counter electrode and a Hg/HgO reference electrode. The reference electrode was calibrated before and after each experiment using an RHE (clean Pt wire in H_2_-saturated electrolyte). This calibration to 0.00 V vs. RHE was, reproducibly, ~−0.87 V vs. Hg/HgO. The Ag-Pd thin films deposited on glassy carbon disks were mounted in a Teflon RDE tip (Pine Research Instruments) and used as working electrodes. We used 0.1 M KOH as the electrolyte, made from semiconductor grade pellets (Sigma Aldrich) and Millipore water (*R* = 18 MΩ cm). All polarization measurements were corrected for the uncompensated electrolyte resistance and background non-ORR contributions measured immediately after the polarization measurements. The activity for the ORR was evaluated by CV at 20 mV s^−1^ from 1 to 0 V vs. RHE. Using thin films <36 hrs old, we performed three CV cycles with continuous oxygen bubbling in O_2_-saturated electrolyte, followed by two CVs with continuous nitrogen bubbling in N_2_-saturated electrolyte to correct for non-ORR currents. Cyclic voltammograms, from 0.7 to 0.5 V vs. RHE, in the same N_2_-saturated electrolyte were collected at 10, 20, 50, 100, and 200 mV s^−1^ to estimate the double-layer capacitance of the thin films to use as a measure of an electrochemically active surface area. With the same RDE setup (1600 rpm, O_2_- or N_2_-sparged 0.1 M KOH), the uncompensated electrolyte resistance was obtained before and after cyclic voltammetry measurements by potential electrochemical impedance spectroscopy.

### Computational methods

#### Density functional theory

Periodic spin−polarized DFT calculations were performed using the RPBE exchange correlational functional^59^, a plane−wave basis set with a cut−off kinetic energy of 500 eV, and the projector-augmented wave method as implemented in the Vienna Ab-initio Simulation Package (VASP version 5.4.4)^60,61^. Pure Ag and Pd surfaces were modeled as five−layer p(3 × 3) (111) fcc slabs with RPBE optimized lattice constants of 4.219 Å and 3.989 Å, respectively. The Brillouin zone was sampled with a (3 × 3 × 1) Monkhorst−Pack grid^62^. Ag-Pd systems were modeled as five−layer p(2 × 2) (111) fcc slabs with optimized lattice constants of Ag_3_Pd_1_ (4.161 Å), Ag_2_Pd_2_ (4.104 Å), and Ag_1_Pd_3_ (4.047 Å) and the Brillouin zone was sampled with a Γ-centered (4 × 4 × 1) Monkhorst−Pack grid. In all of the slabs, the bottom three layers were constrained at the bulk positions, whereas the top two layers and the adsorbed species were fully relaxed. The slabs were separated in the perpendicular z direction by 15 Å of vacuum, and a dipole correction was applied. The electronic convergence criterion was 10^−5^ eV, whereas the force criterion for geometry relaxation was 0.05 eV Å^−1^ for all forces.

Catalytic activities of the different surface structures were evaluated based on the thermodynamic limiting potential for ORR (*U*_*L*_), versus the computational hydrogen electrode (CHE), defined as the highest potential at which all the reaction steps become exergonic by assuming an association reaction mechanism with *OOH, *O, and *OH as reaction intermediates^54,55^. The adsorption free energies, *ΔG*_*x*_ (for *x* = *OOH, *O, and *OH) of the adsorbates are calculated as *ΔG* = *ΔE* + *ΔZPE* − *TΔS*, where *ΔE* is the difference in electronic energy, *ΔZPE* is the difference in zero-point energies, and *ΔS* is the change in entropy of the adsorbed species with respect to the catalyst surface, H_2_(g), and H_2_O(g). The (*ΔZPE* − *TΔS*) value was taken from an earlier publication^54^ and subsequently added to all the adsorption calculations. The difference between the equilibrium potential of *U* = 1.23 V and the limiting potential is referred as the theoretical overpotential (*η*), i.e., *η* = 1.23 V − *U*_*L*_. (See Supplementary DFT Methodology Notes for further details about our DFT methods).

#### FEFF XANES simulations

Pd L_3_-edge XANES simulations were obtained by FEFF 9.9^63^ using the RPA screening approximation and Hedin-Lundquist exchange-correlation potential for an SCF calculation with a cluster of 7 Å, whereas the size of the cluster for the FMS calculation was 9 Å^63^. Convergence was checked both for SCF (from 5 Å to 8 Å) and for FMS (from 6 Å to 10 Å) and a total broadening of 3 eV was used. In order to take into account both the crystal structure expansion and the Ag:Pd atomic ratio, the model for the simulation consists of 1 × 1 × 5 supercells built from the experimental fcc structures for Pd (*a* = 3.8720 Å; ICSD-64918), Ag_0.1_Pd_0.9_ (*a* = 3.911 Å; ICSD-605664), Ag_0.2_Pd_0.8_ (*a* = 3.933 Å; ICSD-197455), Ag_0.5_Pd_0.5_ (*a* = 4.029 Å; ICSD-181301), and Ag_0.8_Pd_0.2_ (*a* = 4.038 Å; ICSD-197458) where the Ag:Pd atomic ratio was adjusted accordingly.

## Supplementary information

Supplementary Information

## Data Availability

All raw data plotted in this work can be accessed on figshare.com through DOI 10.6084/m9.figshare.13049321. Source data are provided with this paper.

## Source data

Source Data
