# Supplementary material for: Tuning the electronic structure of Ag-Pd alloys to enhance performance for alkaline oxygen reduction
Source: Nat Commun. 2021 Jan 27;12:620. doi: 10.1038/s41467-021-20923-z (PMC7840808; doi:10.1038/s41467-021-20923-z)
Supplement: Supplementary file 1 — Supplementary Information [file 41467_2021_20923_MOESM1_ESM.pdf]

Tuning the Electronic Structure of Ag-Pd Alloys to Enhance Performance for Alkaline Oxygen Reduction

Supplementary Information

José A. Zamora Zeledón[1,2]; Michaela Burke Stevens[1,2]; G.T. Kasun Kalhara Gunasooriya[3]; Alessandro Gallo[1,2]; Alan T. Landers[2,4]; Melissa E. Kreider[1,2]; Christopher Hahn[1,2]; Jens K. Nørskov[3] and Thomas F. Jaramillo[1,2,*]

1. Department of Chemical Engineering, Stanford University, 443 Via Ortega, Stanford, California 94305, United States
2. SUNCAT Center for Interface Science and Catalysis, SLAC National Accelerator Laboratory, 2575 Sand Hill Road, Menlo Park, California 94025, United States
3. Catalysis Theory Center, Department of Physics, Technical University of Denmark, DK–2800 Kongens, Lyngby, Denmark
4. Department of Chemistry, Stanford University, 333 Campus Drive, Stanford, California 94305, United States

* Corresponding Author: jaramillo@stanford.edu

Table of Contents

Supplementary Note 1: Grazing Incidence XRD Scans for All Ag-Pd Compositions **2**

Supplementary Note 2: Symmetric XRD Scans for All Ag-Pd Compositions As-Deposited **4**

Supplementary Note 3: Survey XPS Spectra and Compositional Quantification for All Ag-Pd Thin Films Representative of Pre- and Post- Electrochemically Tested Samples **5**

Supplementary Note 4: Full XANES Pd L_3-edge Scan **7**

Supplementary Note 5: AFM Imaging for All Ag-Pd Compositions **8**

Supplementary Note 6: Electrochemical ORR Activity Evaluation at All Ag-Pd Compositions **12**

- Characteristic CV profiles under N_2-staturated electrolyte **15**
- Koutecký–Levich Analysis **18**
- Tafel Analysis **18**
- Performance Summary **19**

Supplementary Note 7: Kinetic Current Normalizations **20**

Supplementary Note 8: Stability/Durability Analysis **22**

- Representative Stability Testing of $Ag_{0.1}Pd_{0.9}$ **23**
- Limited $Ag_{1-x}Pd_x$ Stability Testing **23**

Supplementary Note 9: Comparison to State-of-the-Art Specific Activity **25**

Supplementary Note 10: Density Functional Theory Modeling **27**

Supplementary Information References **32**

Supplementary Note 1: Grazing Incidence XRD Scans for All Ag-Pd Compositions

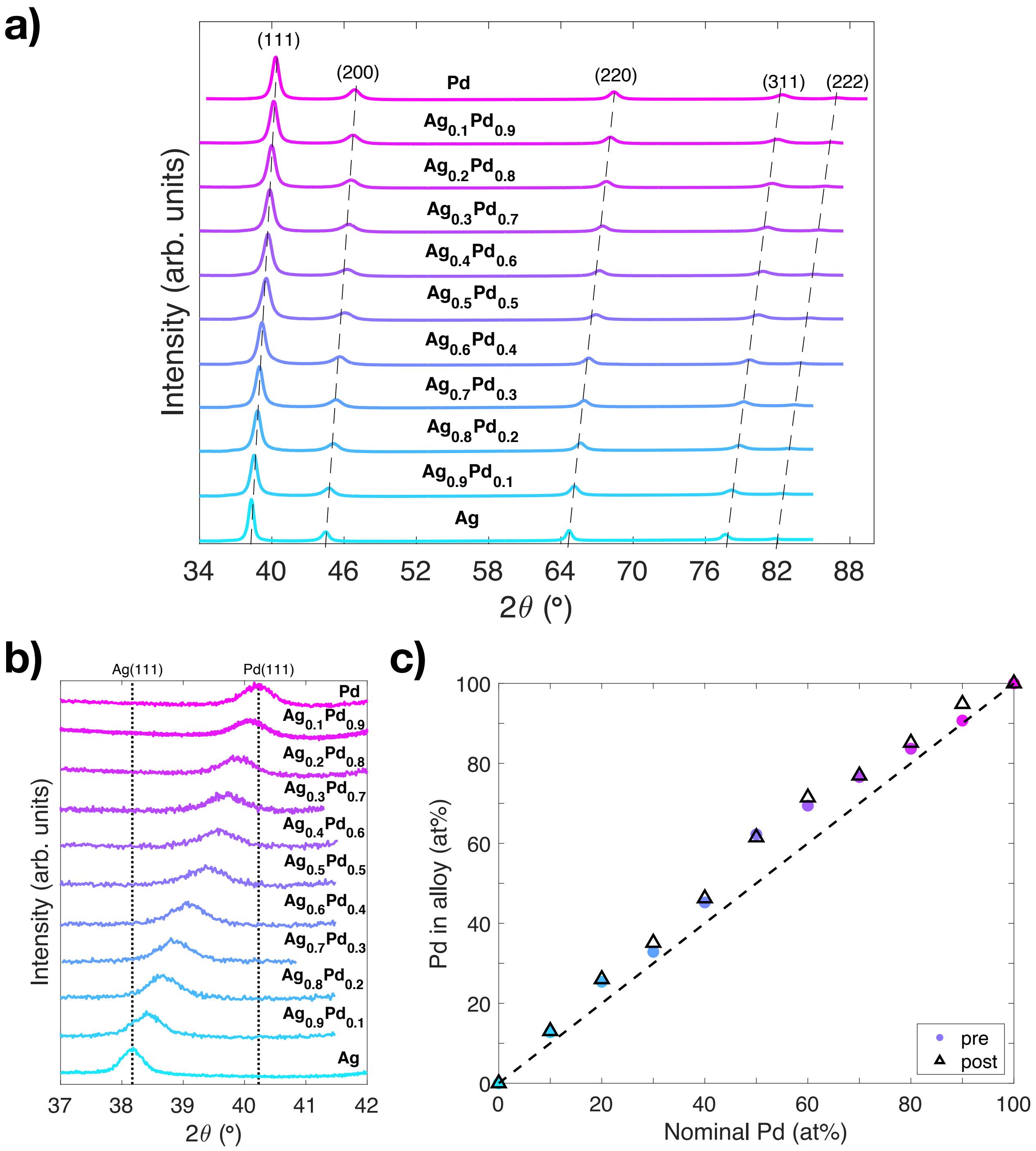

Supplementary Figure 1. GI-XRD of Ag-Pd thin films. (**a**) Full grazing incidence (GI) x-ray diffractograms of as-prepared Ag, Pd, and mixed $Ag_{1-x}Pd_{1-x}$ thin films (deposited on standard microscope glass slides). Full 2θ range for data shown in **Figure 1a**. (**b**) GI x-ray diffractograms of the (111) peak for Ag, Pd, and Ag-Pd thin films of different composition after ORR testing, with Ag (38.17°) and Pd (40.23°) peak locations shown by dotted lines, and (**c**) Vegard's Law analysis of the (111) peak showing Pd content in alloy versus nominal Pd composition both pre- and post- electrochemical ORR activity measurements. All post-electrochemistry characterization was done with thin films deposited on glassy carbon substrates.

Grazing incidence XRD measurements on the thin films after electrochemical testing (**Supplementary Figure 1b**), along with the corresponding Vegard's Law analysis (**Supplementary Figure 1c**), indicates that the structure and alloy composition of the thin films did not change significantly after electrochemical experiments. The minor differences in alloy composition given by Vegard's Law before and after electrochemistry are likely due to the low signal to noise ratio in the post-ORR testing diffractograms of the thin films taken on glassy carbon substrates. The absolute value differences in 2θ (111) peak position of the as-deposited versus the after electrocatalysis seen in **Supplementary Table 1** are likely due to the different substrates used, as well as the different grazing incidence angle needed to obtain a measurable signal when using glassy carbon substrates.

Supplementary Table 1. 2θ (111) peak position, d_{111}-spacing, and lattice constant of all Ag-Pd thin film compositions based on GI-XRD measurements taken before (blue) and after (green) electrochemical testing. (In reference to **Figure 1a** and **Supplementary Figure 1**).

	As-deposited on glass slides			Post electrocatalysis on glassy carbon		
**Nominal Pd (at%)**	**(111) peak 2θ position (°)**	**d_{111} (Å)**	**a (Å)**	**(111) peak 2θ position (°)**	**d_{111} (Å)**	**a (Å)**
**0**	38.30	2.35	4.07	38.17	2.36	4.08
**10**	38.55	2.33	4.04	38.42	2.34	4.06
**20**	38.80	2.32	4.02	38.68	2.33	4.03
**30**	38.95	2.31	4.00	38.87	2.32	4.01
**40**	39.20	2.30	3.98	39.09	2.30	3.99
**50**	39.55	2.28	3.94	39.41	2.29	3.96
**60**	39.70	2.27	3.93	39.62	2.27	3.94
**70**	39.85	2.26	3.92	39.73	2.27	3.93
**80**	40.00	2.25	3.90	39.91	2.26	3.91
**90**	40.15	2.24	3.89	40.12	2.25	3.89
**100**	40.35	2.23	3.87	40.23	2.24	3.88

Supplementary Note 2: Symmetric XRD Scans for All Ag-Pd Compositions As-Deposited

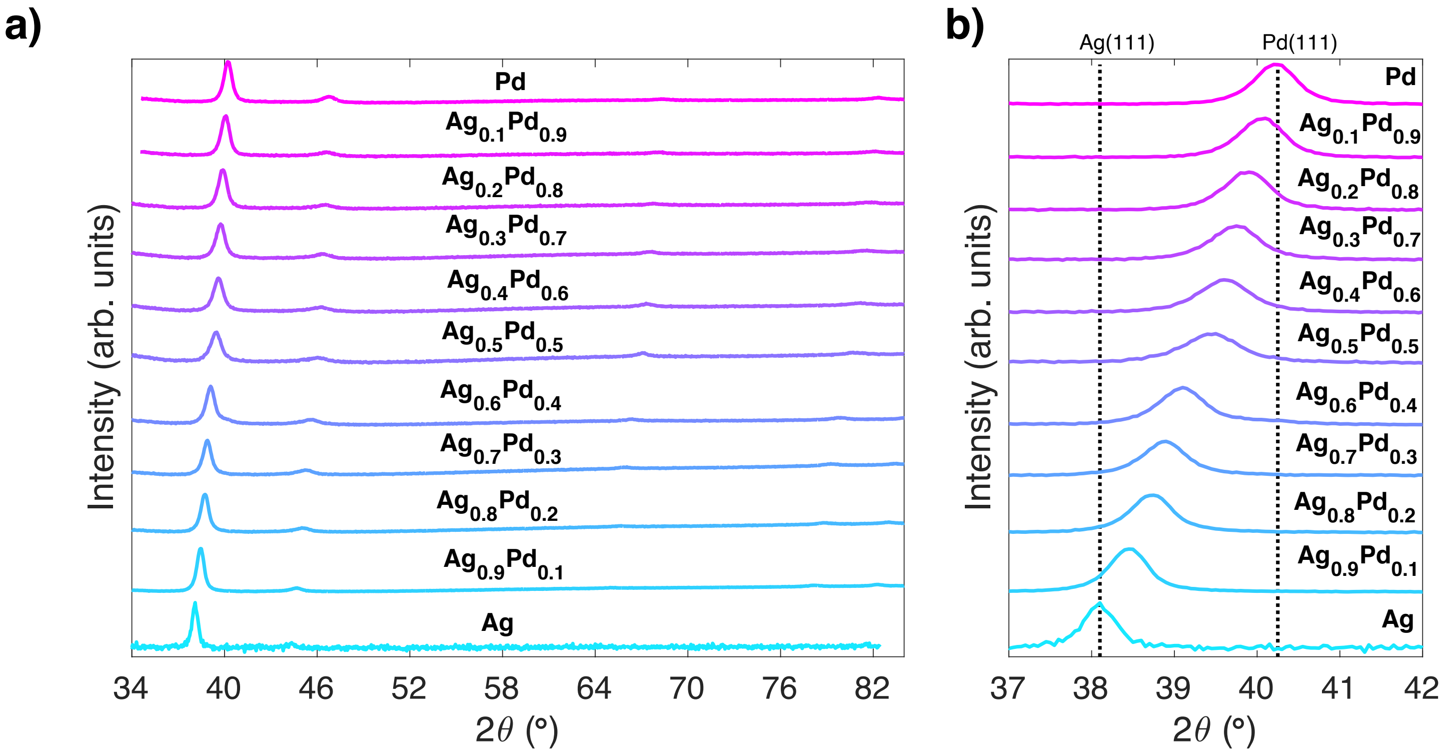

Supplementary Figure 2. Symmetric XRD of Ag-Pd thin films. (**a**) Full-scan symmetric x-ray diffractograms and (**b**) magnified symmetric x-ray diffractograms at the (111) peak with Ag (38.1°) and Pd (40.25°) peak locations shown by dotted lines for Ag, Pd, and Ag-Pd thin films of different composition deposited on standard microscope glass slides. (Relevant for **Figure 1a** in main text).

Similarly to in GI-XRD, a strong (111) peak is also present in all diffractograms taken in a symmetric scan (ω = half of 2θ range) geometry (**Supplementary Figure 2**), consistent with a preferential ⟨111⟩ out of plane orientation.[1,2] Specifically, the intensity ratio of the (200) to (111) peak is about ~ 0.25 – 0.3, whereas in the reference computed XRD patterns[3,4] this ratio is ~ 0.44-0.48. This preferential orientation has been noted previously and attributed to the thermodynamically favorability of fcc metals to grow with their {111} facet exposed.[1,2]

Supplementary Note 3: Survey XPS Spectra and Compositional Quantification for All Ag-Pd Thin Films Representative of Pre- and Post- Electrochemically Tested Samples

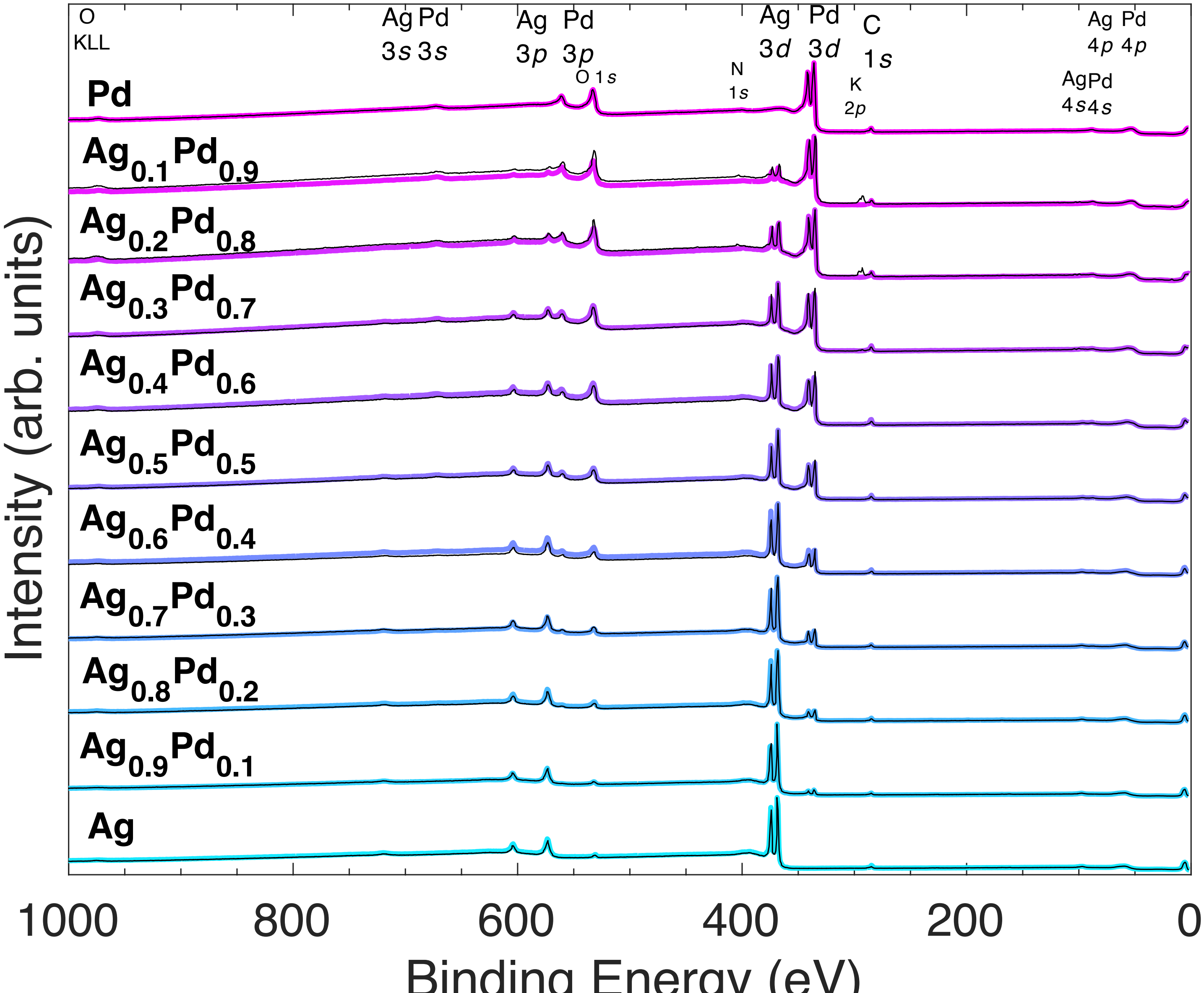

Supplementary Figure 3. Full-scan survey (Su) XPS spectra for Ag, Pd, and Ag-Pd thin films of different composition representative of samples pre- ("cool" color map) and post- (black) electrochemical ORR testing. The pre- and post-ORR Su spectra look nearly identical with the only notable differences being slightly different background intensities and small K 2*p* and N 1*s* peaks specifically for the post-ORR 80 and 90 at% Pd content samples. Any potassium signal likely comes from trace/residual potassium on the sample surface due to being submerged in 0.1 M KOH or from trace potassium accumulated due to storage and transport in air. Any nitrogen signal likely comes from minor contamination due to air storage and transport and/or trace N_2 inside the XPS chamber. The C 1*s* peak is set to 284.8 eV for all spectra. Peaks are labeled at the top. At ~530 eV there is a small O 1*s* peak for Ag; and for the other spectra it overlaps with the Pd $3p_{3/2}$ peak. (Relevant for **Figures 1b** and **1c** in main text).

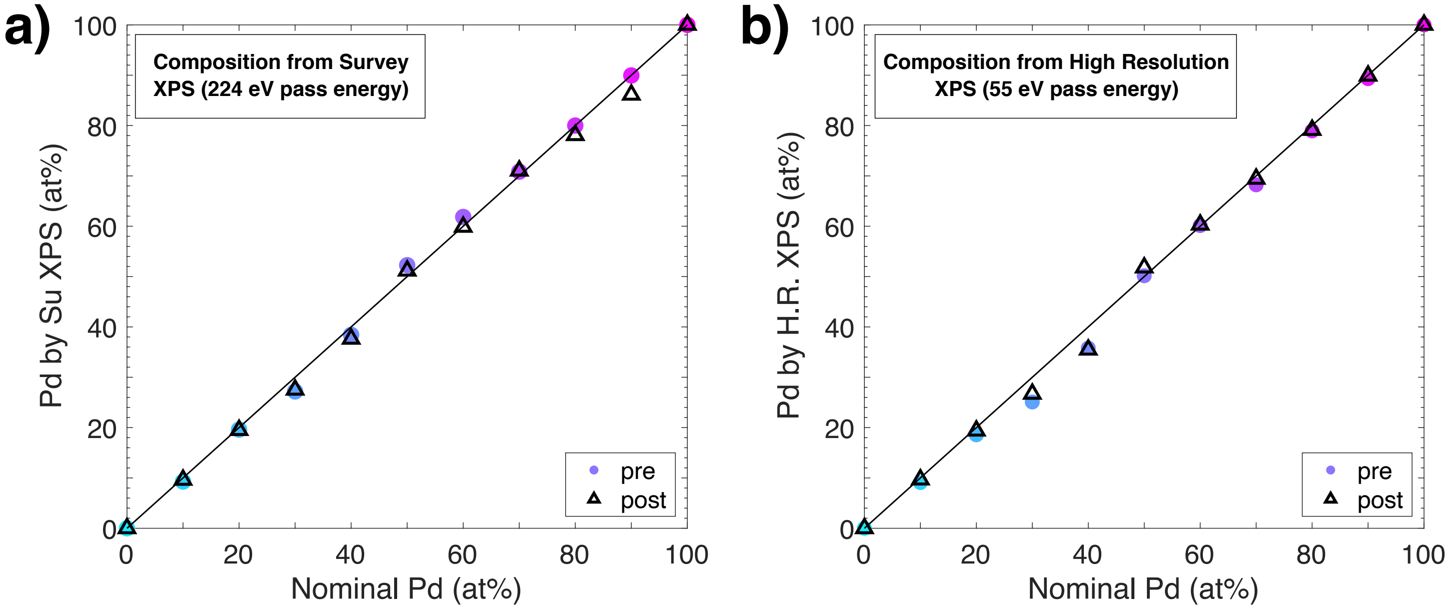

Supplementary Figure 4. Near-surface composition determined by XPS pre- (circles) and post- (triangles) electrochemical testing for Ag, Pd, and Ag-Pd thin films of different composition determined from (**a**) survey and (**b**) high resolution XPS spectra.

A compositional analysis from XPS measurements (**Figures 1c, 1d,** and **Supplementary Figure 3**) taken before and after electrochemical ORR testing indicates that the thin films exhibited surface compositions consistent with nominal bulk values both pre- and post- electrochemical ORR testing (**Supplementary Figure 4**). This suggests, that within instrument sensitivity, the near-surface composition of the thin films was stable during ORR activity measurements.

Supplementary Note 4: Full XANES Pd L_3-edge Scan

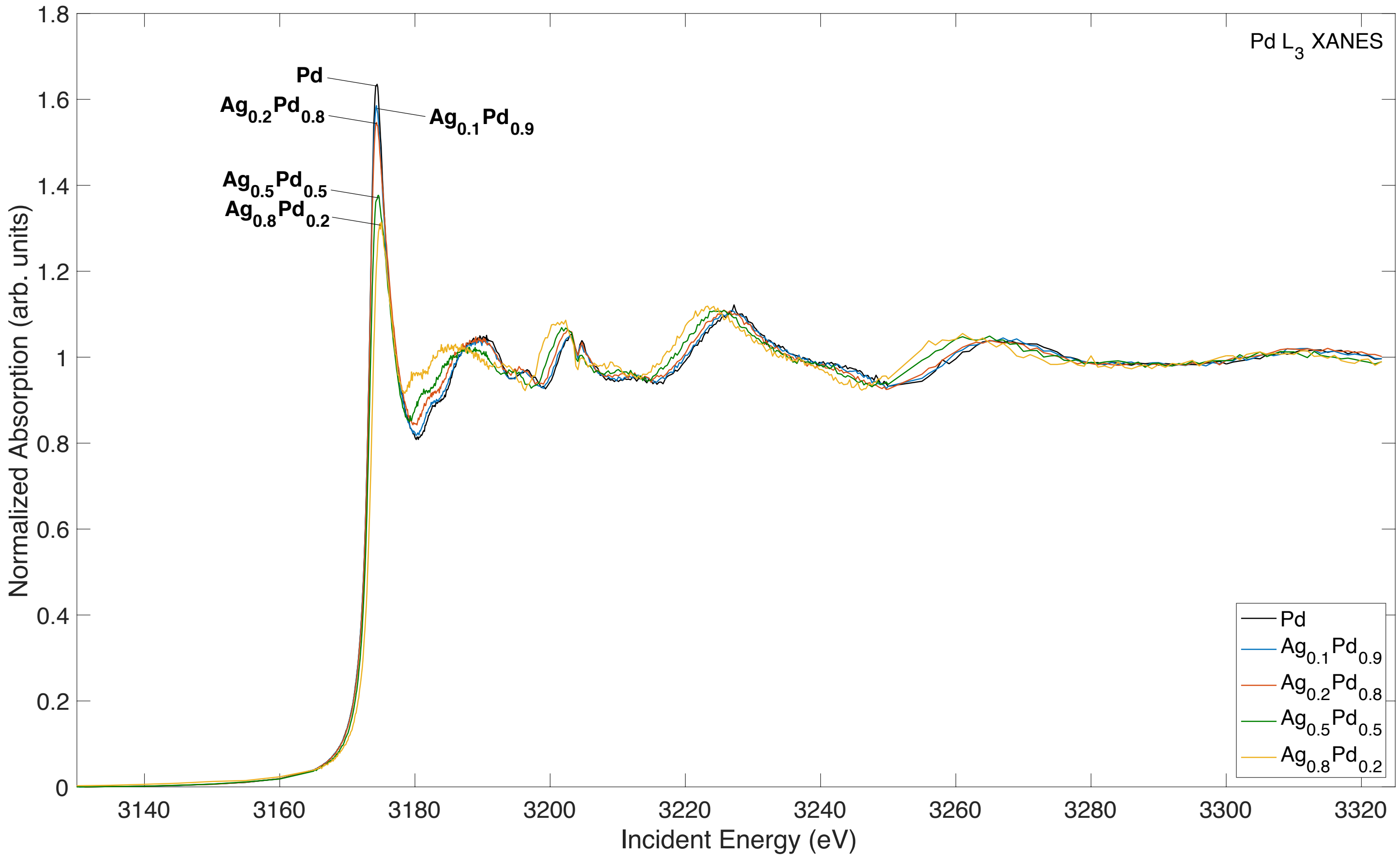

Supplementary Figure 5. Full-scan Pd L_3 x-ray absorption near edge spectra for Pd, $Ag_{0.1}Pd_{0.9}$, $Ag_{0.2}Pd_{0.8}$, $Ag_{0.5}Pd_{0.5}$, $Ag_{0.8}Pd_{0.2}$. All samples measured on glassy carbon substrates. Full energy-range XAS spectra for data shown in **Figure 1e**.

The filling in the Pd 4*d*-band results in an increase in the incident energy needed to excite Pd electrons from the 2*p* to 4*d* band, which is illustrated by the positive energy shifts (ranging between 0.5 – 1 eV) in the white-line position with increased Ag content.[5,6]

As explained in the main manuscript, our Pd L_3 XANES agrees wells with literature, and therefore we hypothesize that the valence band and unoccupied *d*-states of our Ag-Pd alloys should follow the trends previously observed in literature for related systems.[7]

Supplementary Note 5: AFM Imaging for All Ag-Pd Compositions

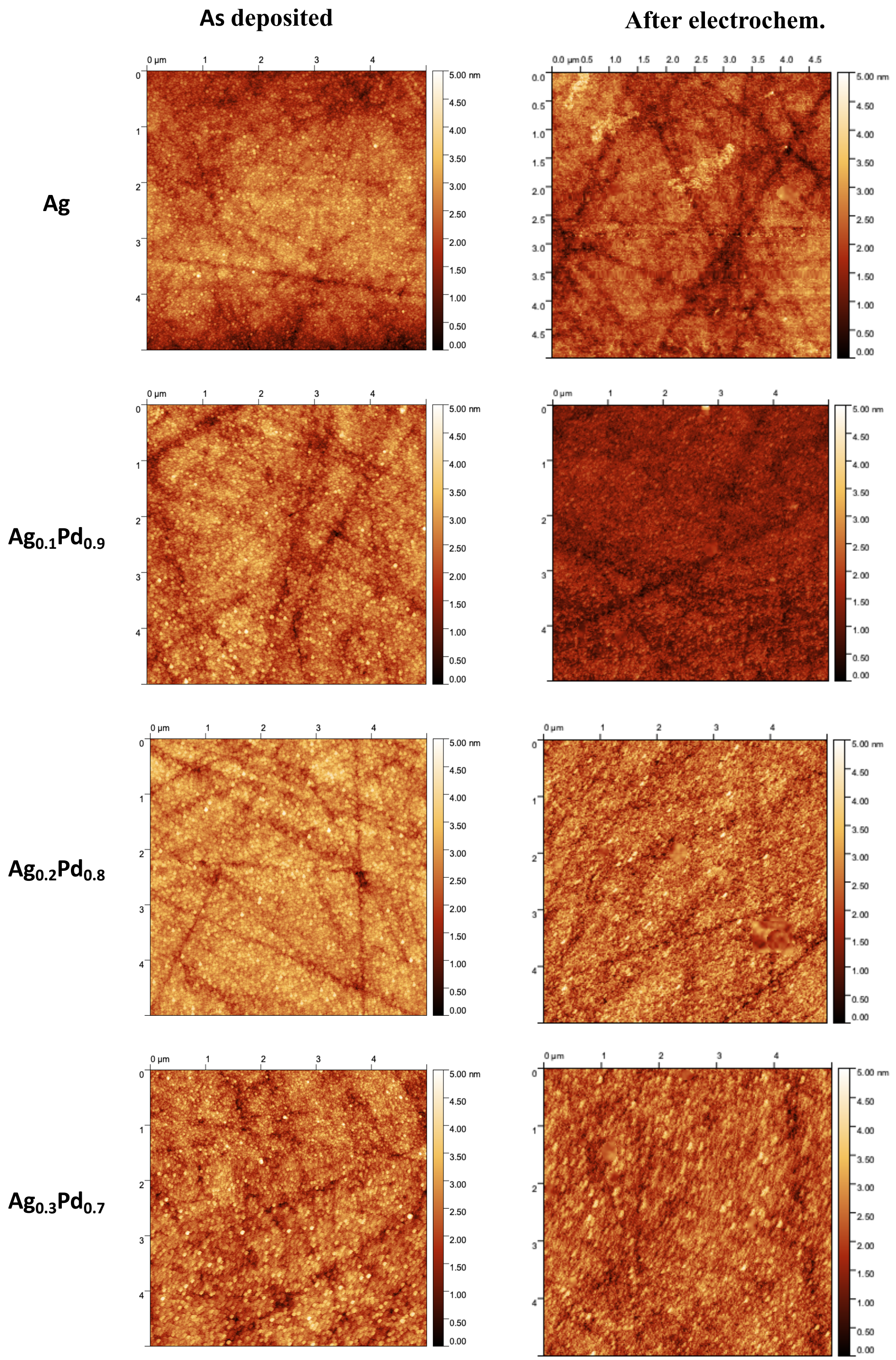

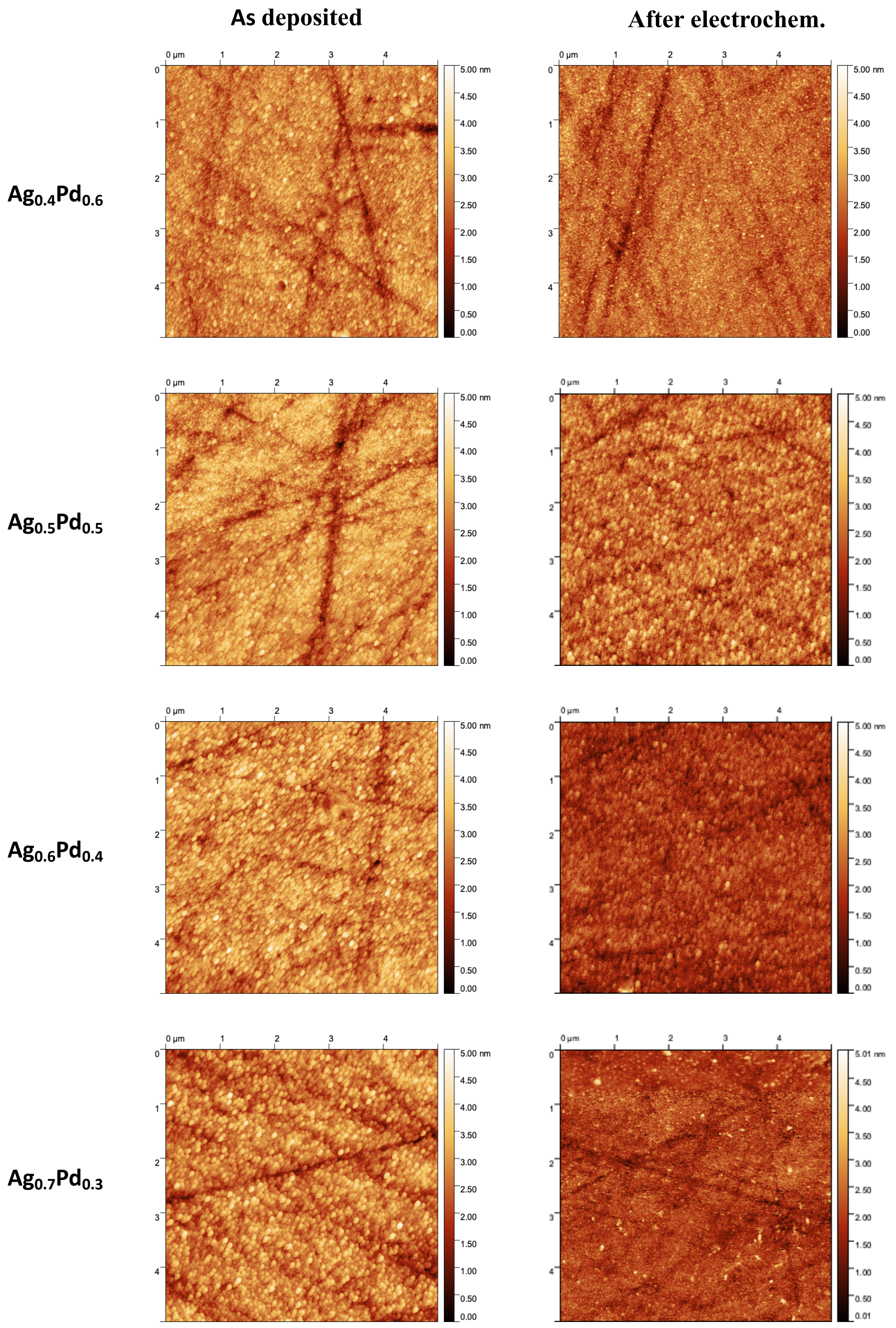
As deposited
After electrochem.
Ag0.4Pd0.6
Ag0.5Pd0.5
Ag0.6Pd0.4
Ag0.7Pd0.3
5.00 nm
0.00

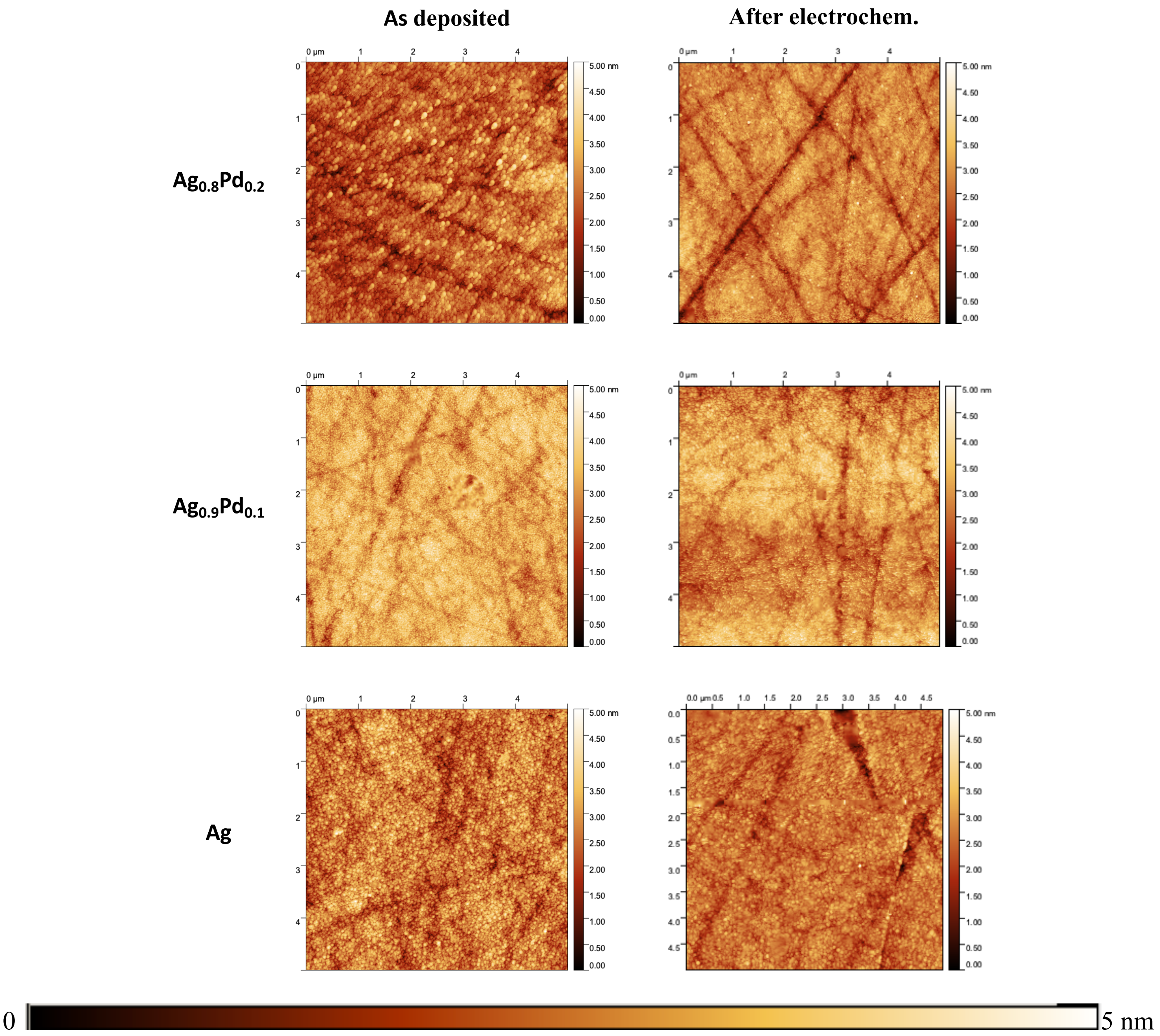

Supplementary Figure 6. AFM topography images before and after ORR activity testing for Ag, Pd, and Ag-Pd thin films of different composition deposited on glassy carbon substrates. “After electrochem.” means: after 3 ORR CVs followed by 2 N_2 CVs at 20 mV s^{-1} from 1 – 0 V vs. RHE. Linear color scale from 0 nm to 5 nm (*z* [height]). (Relevant for **Figure 2** in main text).

Flatness/smoothness in AFM topographical images is represented by color homogeneity, not the actual color being displayed. For example, for $Ag_{0.7}Pd_{0.3}$, the “post” image is more dark-red in color compared to the “as

deposited” image, but both images are highly homogeneous in their own color distribution, meaning that they are both highly smooth/flat. The difference in the main/most noticeable color among images can be due to differences in the magnitude of the interactions between the cantilever and the sample surface, as well as the scanning settings, which change sample to sample.

AFM was used to determine the surface roughness and topography of the thin films before and after electrochemical testing. AFM imaging shows a lack of topographical features on the thin films both before and after electrochemical measurements (**Supplementary Figure 6**). The surface roughness factors (RF) of the thin films were estimated by AFM imaging (**Supplementary Table 2**), and they suggest the thin films are very flat and remain flat after electrochemical experiments with RFs ≤ 1.02.

Supplementary Table 2. Roughness factor (RF) and root mean squared (RMS) roughness calculated by AFM for all thin film compositions studied both as deposited and after electrochemical testing.

sample		as deposited		post electrochemical testing	
nominal Pd%	nominal Ag%	RF	RMS roughness (nm)	RF	RMS roughness (nm)
**100**	**0**	1.014	3.625	1.005	2.084
**90**	**10**	1.013	2.600	1.023	3.609
**80**	**20**	1.010	2.356	1.017	3.755
**70**	**30**	1.011	2.195	1.009	2.360
**60**	**40**	1.009	2.158	1.023	2.378
**50**	**50**	1.008	2.339	1.009	2.411
**40**	**60**	1.005	1.779	1.009	2.429
**30**	**70**	1.005	2.231	1.019	2.957
**20**	**80**	1.007	2.377	1.007	2.598
**10**	**90**	1.017	2.386	1.009	3.010
**0**	**100**	1.018	2.874	1.012	5.946

Supplementary Note 6: Electrochemical ORR Activity Evaluation at All Ag-Pd Compositions

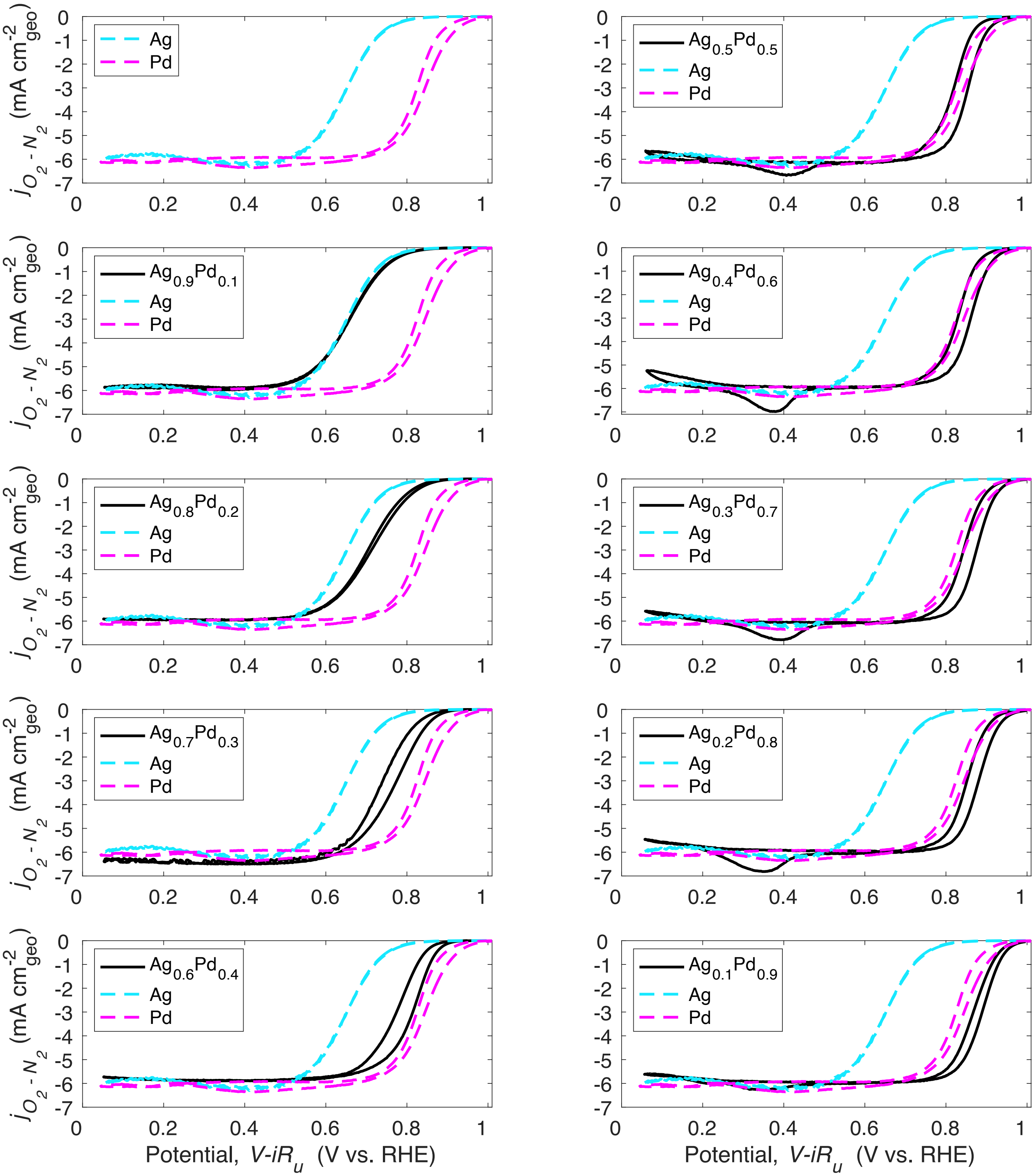

Supplementary Figure 7. Representative 3rd cycle CV curves in O_2-saturated 0.1 M KOH for Ag, Pd, and Ag-Pd thin films of different composition at 1600 rpm and at 20 mV s^{-1}. (Relevant for **Figure 2** in main text). The feature present around 0.2 – 0.4 V vs. RHE for the ≥ 50 at% Pd samples is due to an overcorrection in the HUPD region with the subtraction of the N_2 CV profile, suggesting that HUPD is suppressed during the ORR.

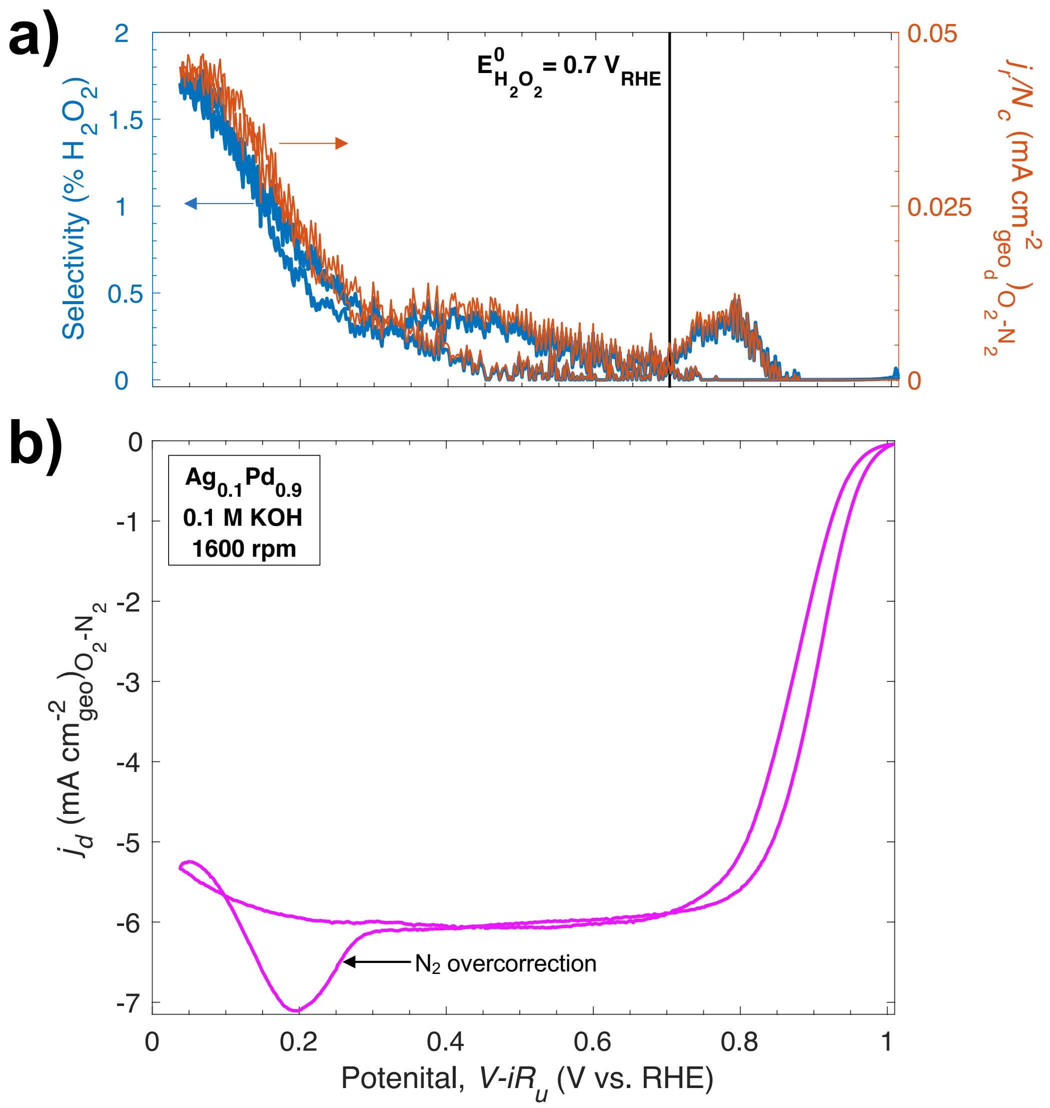

Supplementary Figure 8. Rotating ring disk electrode (RRDE) measurements for $Ag_{0.1}Pd_{0.9}$. (**a**) H_2O_2 selectivity (left, blue) and equivalent baseline corrected H_2O_2 ring current density (right, orange; based on disk area and a 0.20 collection efficiency, N_c), and (**b**) the ORR disk geometric current density as a function of potential. The Pt ring was set at 1.2 V vs. RHE. The ring collection efficiency was determined by calibration against the ferrocyanide/ferricyanide (0.004 M ferricyanide in 0.1 M KOH) redox couple as done by Zhou, et al[8]. A feature in the disk current density arising from an N_2 CV profile overcorrection of HUPD features is preset at around 0.2 V vs. RHE.

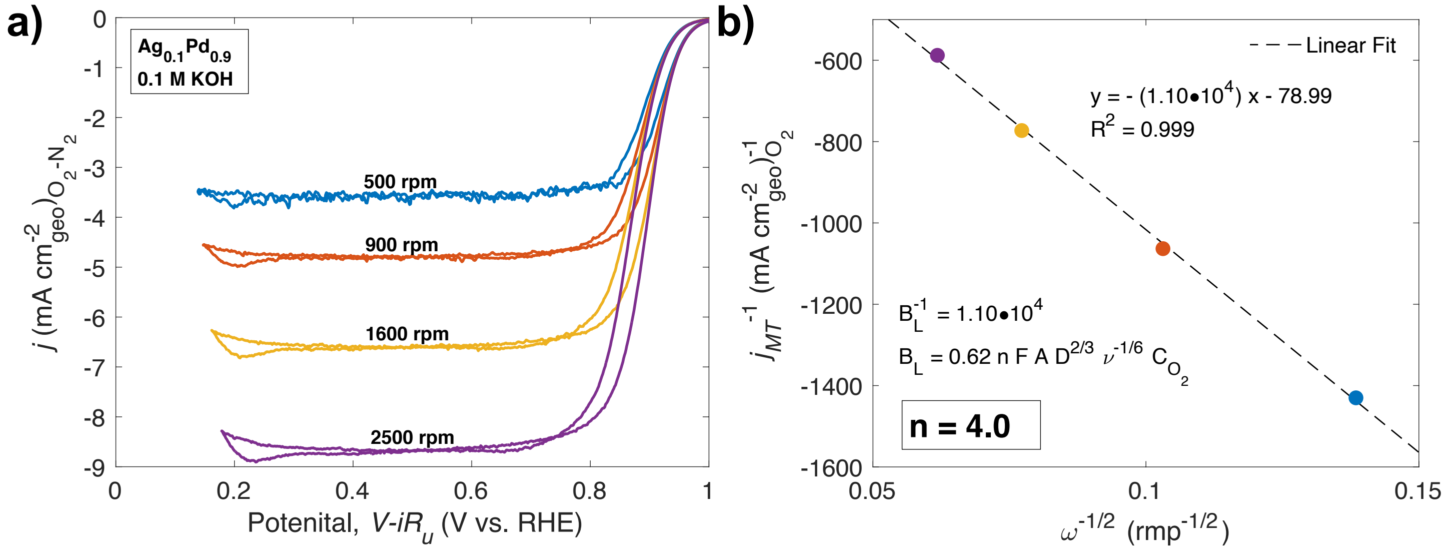

Supplementary Figure 9. Koutecký–Levich analysis. (**a**) ORR CVs as a function of RDE rotation rate, and (**b**) corresponding Koutecký–Levich (KL) selectivity analysis (inverse mass transport (MT) limited current density as a function of the inverse square root of rotation rate) for $Ag_{0.1}Pd_{0.9}$. For this experiment the glassy carbon substrate/disk was slightly taller than the change disk insert cavity, causing the edges of the disk, which had catalyst material on it, to be exposed, and hence slightly increasing the overall catalyst surface area relative to the geometric electrode area (0.196 cm^2) leading to mass transport limited current densities slightly more negative than expected given $4e^-$ selectivity. We used F = 96485.333 C mol^{-1}, A = 0.196 cm^{-2}, D = 1.93 $x10^{-5}$ cm^2 s^{-1}, ν = 1.09 $x10^{-2}$ cm^2 s^{-1}, and C_{O2} = 1.26 $x10^{-6}$ mol cm^{-3}.[9]

In addition to suggesting full $4e^-$ selectivity, the KL measurements in **Supplementary Figure 9** have overlapping kinetically controlled regions, indicating that mass transport limitations were not problematic.

Characteristic CV profiles under N_2-staturated electrolyte

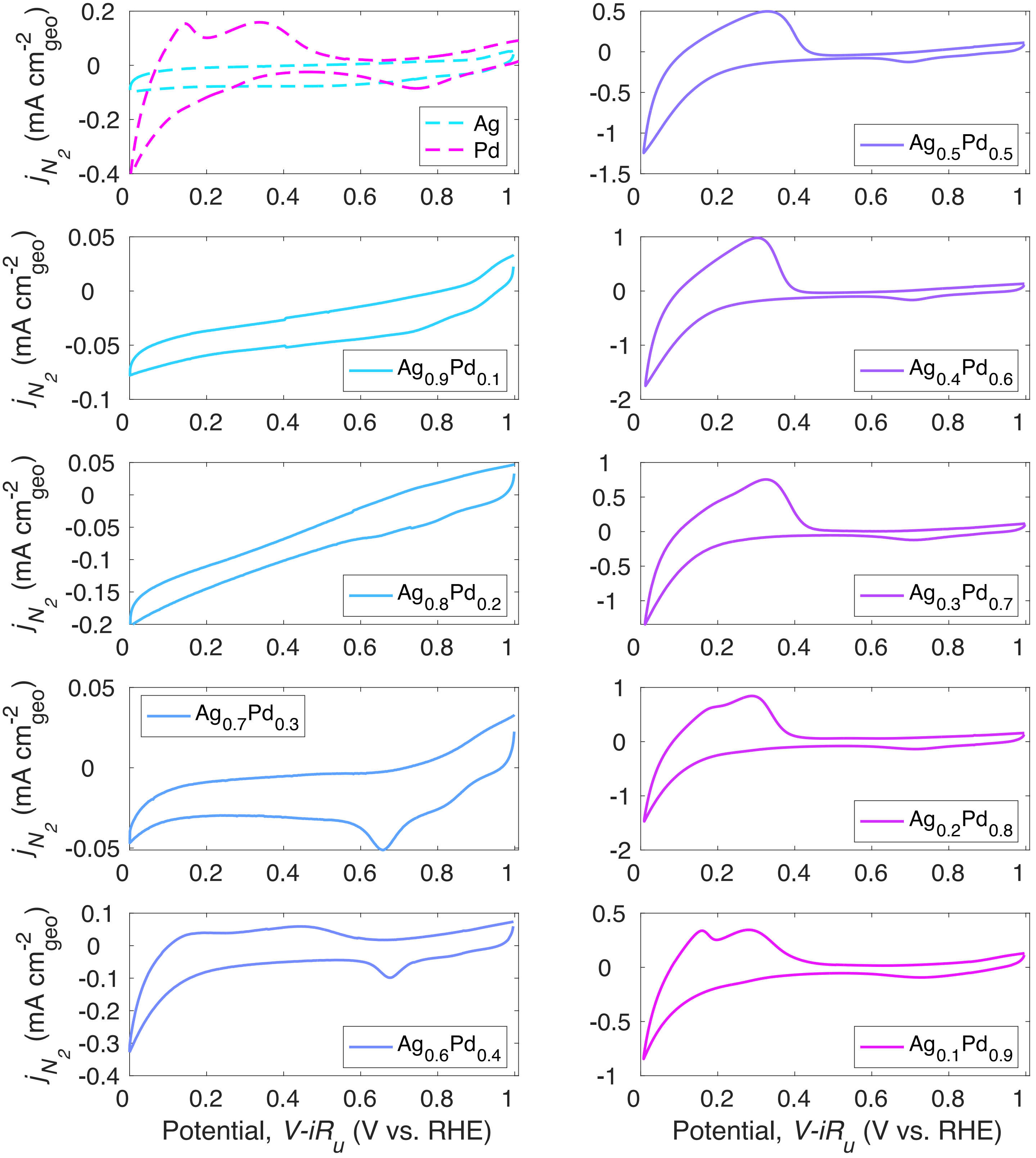

Supplementary Figure 10. Representative 2nd cycle CV curves in N_2-saturated 0.1 M KOH for Ag, Pd, and Ag-Pd thin films of different composition at 1600 rpm and at 20 mV s^{-1}. (Relevant for **Figure 2** in main text).

The CV profiles in N_2-saturated electrolyte for all $Ag_{1-x}Pd_x$ compositions investigated here are shown in **Supplementary Figure 10**. The CV profile for pure Ag has similar redox features as studied previously on single crystals and polycrystalline electrodes.[10–13] Redox features around 0.3 to 0.5 V vs. RHE arise from the adsorption and desorption of loosely bound hydroxide species. The lack of a major Ag reduction and oxidation features suggests the electrode remained mostly metallic for the entire scan. The CV profile for pure Pd has similar redox features as investigated previously on Pd electrodes.[14,15] The Pd CV exhibits a convoluted feature for hydrogen intercalation (expected negative of 0.1 V vs. RHE) and surface adsorption in the cathodic scan around 0 to 0.4 V vs. RHE, with corresponding hydrogen oxidation features in the anodic scan.[14,15] The lack of a major Pd oxidation feature in the anodic scan suggests that an extensive oxide layer is not forming at the surface, and that the surface remains mostly metallic. The small Pd reduction peak (compared to previous work[14–18]) around 0.75 V vs. RHE further supports this hypothesis. The CV profiles for the $Ag_{1-x}Pd_x$ thin films have a combination of the Ag and Pd features. The Pd reduction feature becomes noticeable at ≥30 at% Pd loading and shifts to slightly higher potentials with increasing Pd content (approximately from 0.65 to 0.75 V vs. RHE). The reversible Pd hydrogen intercalation and surface adsorption features become noticeable at a ≥40 at% Pd loading and are most intense for $Ag_{0.4}Pd_{0.6}$. The changes in the magnitude of the reversible hydrogen surface adsorption redox features among the profiles of the bimetallic samples with ≥50 at% Pd suggest that these processes become easier when the Pd lattice is expanded due to the addition of Ag.

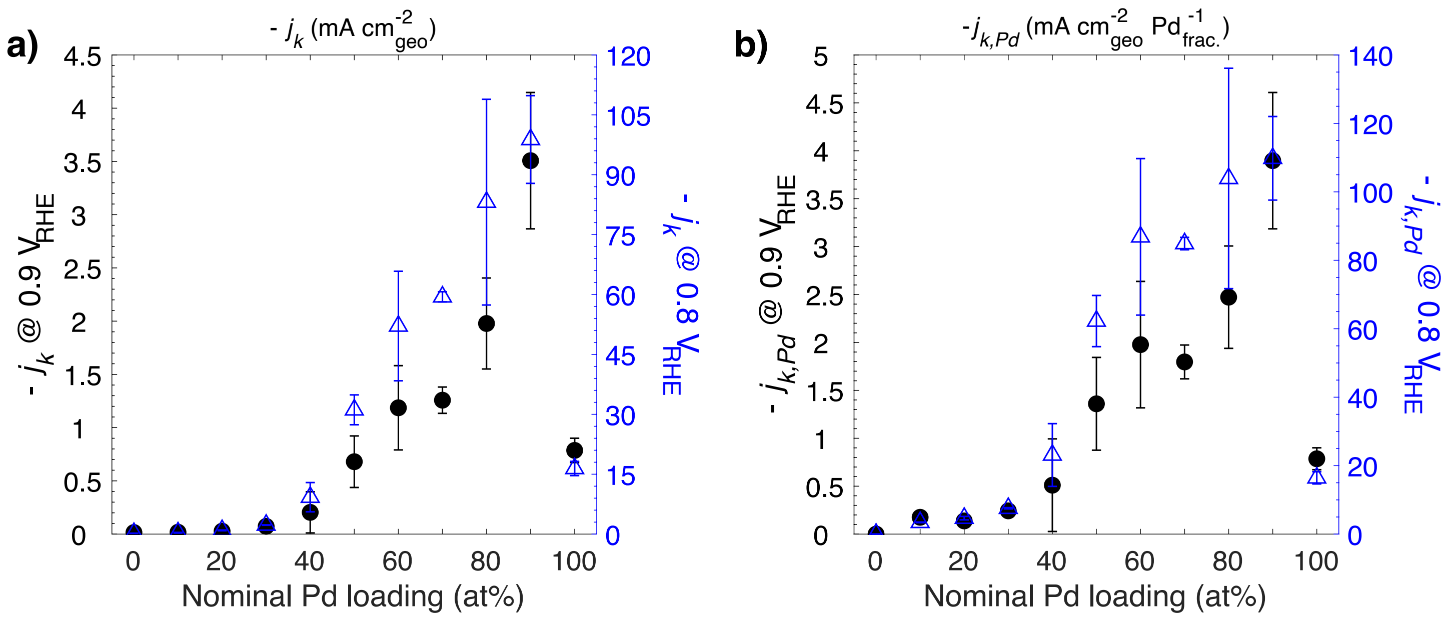

Supplementary Figure 11. Geometric kinetic current density for Ag-Pd thin films. (**a**) Kinetic current density at 0.9 V (left, black circles) and 0.8 V (right, blue triangles) vs. RHE, and (**b**) kinetic current density normalized by Pd loading at 0.9 V (left, black circles) and 0.8 V (right, blue triangles) vs. RHE. All data points are representative of the average value from the cathodic and anodic scan of the third CV cycle and error bars are the standard deviation from measurements with separate samples (n = 2 samples for 0–30 at% Pd, and n = 3 for 40–100 at% Pd). (Relevant for **Figure 2c** in main text).

Due to the large activity range between Ag and Pd, two different metrics were used to compare catalysts: current densities at 0.9 V vs. RHE (**Figure 2c - circles**) for the higher performing systems that are competitive with the state-of-the-art catalysts, and current densities at 0.8 V vs. RHE (**Supplementary Figure 11a**) to meaningfully compare among the Ag-rich catalysts (< 50 % Pd) that have lower onset potentials (< 0.92 V vs. RHE). While error in the extrapolation to kinetic current increases as current increases to values similar to the mass transport limited current, we still use 0.8 V vs. RHE as a reference potential for our best-performing samples to enable a fair constant potential comparison of activity among all the samples investigated in this work (the ORR on the samples with ≤30 at% Pd onsets at potentials lower than 0.9 V vs. RHE, making kinetic data at this potential less meaningful for these compositions). Therefore, extracting and comparing kinetic current densities at both 0.9 V and 0.8 V vs. RHE provides the best way to compare kinetic data at relevant potentials across samples and across the literature.

Koutecký–Levich Analysis

The Koutecký–Levich equation[19] can be expressed as:

$$\frac{1}{i} = \frac{1}{i_k} + \frac{1}{i_{MT}} \quad \textbf{(Equation 1)}$$

where, i is the experimentally measured current, i_k is the kinetic current from the specific electrochemical reaction, and i_{MT} is the experimentally measured mass transport limited current density. i and i_{MT} can be found using cyclic voltammetry, and then used to solve for i_k. Kinetic current density, j_k, can be obtained by normalizing i_k by a representative catalyst surface area, and thus be used as a measure of specific (intrinsic) activity.

Tafel Analysis

A Tafel analysis consists of plotting the base ten logarithm of activity (in our case kinetic current density) versus potential, where the slope(s) of such curve corresponds to the Tafel slope(s). **Supplementary Figure 12** shows a representative Tafel plot analysis using the ORR CV curves in **Supplementary Figure 7**. The Tafel slope is a measure of the number of volts required to increase activity by one order of magnitude. Tafel slopes are reported in **Figure 2b**.

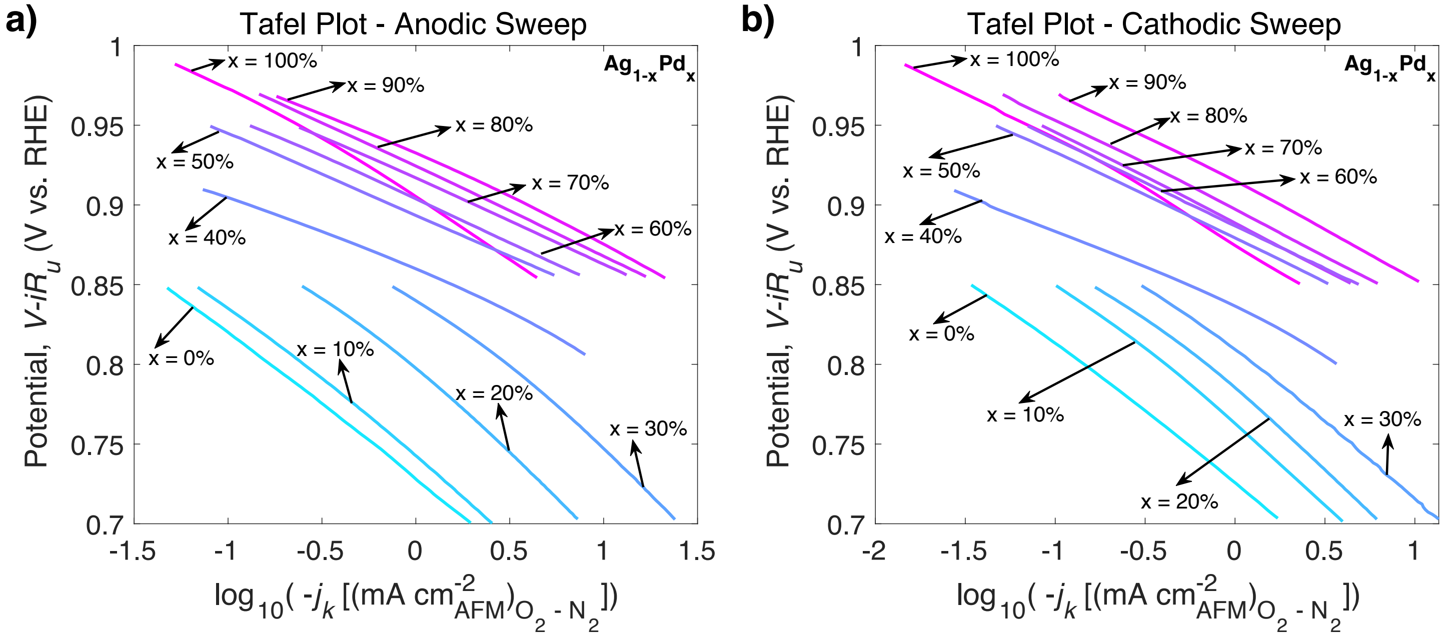

Supplementary Figure 12. Representative Tafel plot analysis (using the ORR CV curves in **Supplementary Figure 7**). (**a**) Anodic of forward sweep and (**b**) cathodic or reverse sweep. (Relevant for **Figure 2** in main text).

Performance Summary

Supplementary Table 3. Summary of $Ag_{1-x}Pd_x$ ORR activity performance. The kinetic current densities are normalized by the roughness factors determined by AFM (**Supplementary Table 2**), and are representative of specific activity. The Tafel Slopes are for the low current density (lcd) regions. Exchange current densities were calculated by setting potential equal to 1.23 V vs. RHE and using the lcd region Tafel linear fit (Potential = Tafel Slope * $\log_{10}(-j_k)$ + constant). All average (AVG) data points are representative of the average value from the cathodic and anodic scan of the third CV cycle from measurements on separate samples (n = 2 samples for 0–30 at% Pd, and n = 3 for 40–100 at% Pd). Relevant for **Figure 2c** in the main text, and **Supplementary Table 4**.

Sample	Onset Potential (V_{RHE}) AVG @0.1 mA cm_{geo}^{-2}	- j_k (mA cm_{AFM}^{-2}) @ 0.9 V vs RHE, AVG	- j_k (mA cm_{AFM}^{-2}) @ 0.9 V vs RHE, Cathodic	- j_k (mA cm_{AFM}^{-2}) @ 0.9 V vs RHE, Anodic	- Tafel Slope (mV dec^{-})AVG lcd	- AVG Kin. Exchange current density (mA cm_{geo}^{-2})
Pd	0.956	0.775	0.416	1.134	57.9	1.51E-06
$Ag_{0.1}Pd_{0.9}$	0.977	3.463	2.024	4.902	51.2	1.10E-06
$Ag_{0.2}Pd_{0.8}$	0.965	1.958	0.924	2.991	48.3	3.09E-07
$Ag_{0.3}Pd_{0.7}$	0.959	1.244	0.586	1.901	44.5	4.00E-08
$Ag_{0.4}Pd_{0.6}$	0.948	1.176	0.480	1.871	41.5	1.10E-08
$Ag_{0.5}Pd_{0.5}$	0.935	0.674	0.266	1.083	39.9	5.23E-09
$Ag_{0.6}Pd_{0.4}$	0.916	0.203	0.094	0.312	45.7	2.95E-08
$Ag_{0.7}Pd_{0.3}$	0.891	0.072	0.047	0.098	89.6	3.91E-05
$Ag_{0.8}Pd_{0.2}$	0.871	0.027	0.028	0.027	97.9	3.52E-05
$Ag_{0.9}Pd_{0.1}$	0.843	0.017	0.017	0.018	94.4	9.24E-06
Ag	0.825	0.015	0.014	0.016	90.3	3.58E-06

Supplementary Note 7: Kinetic Current Normalizations

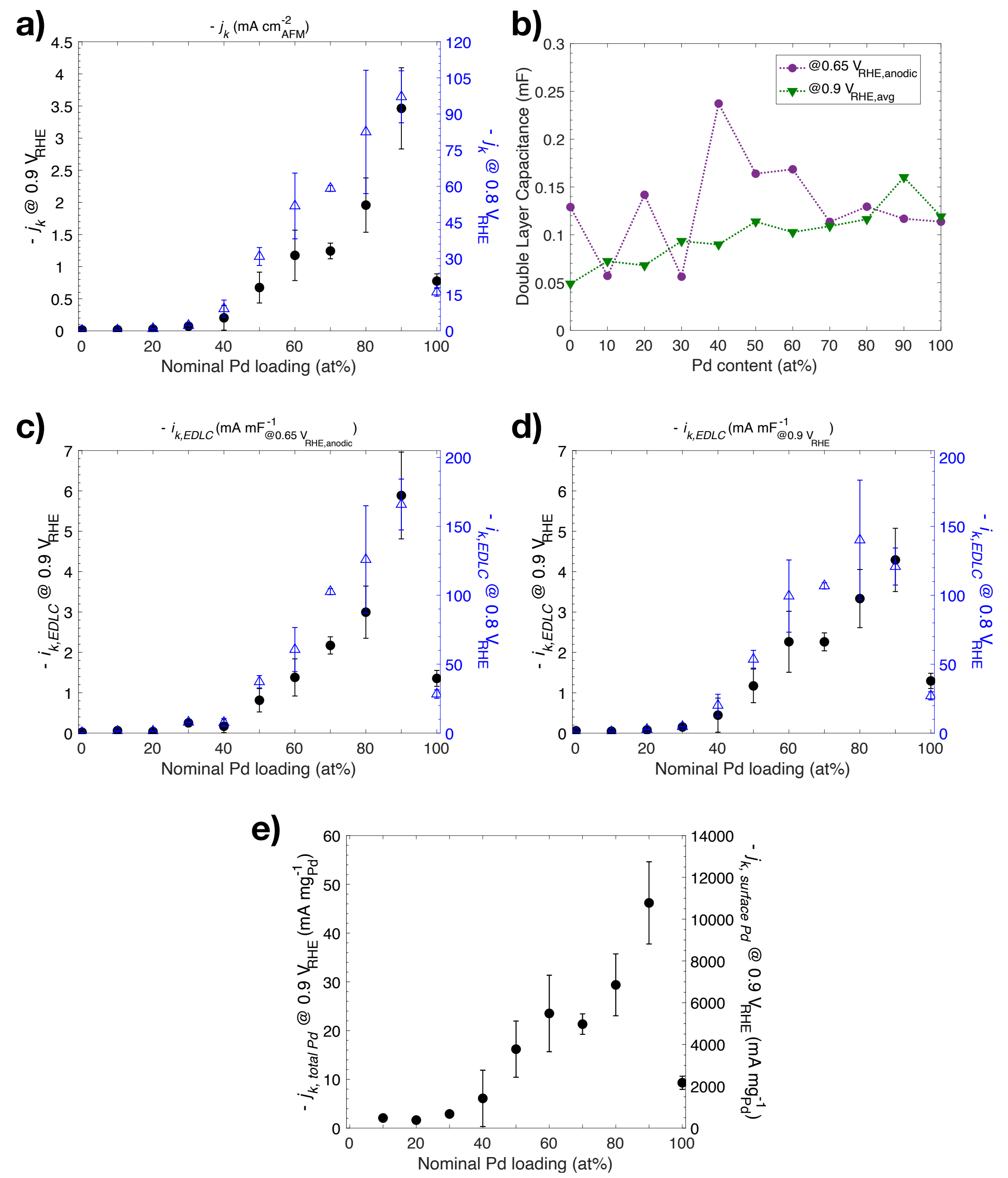

Supplementary Figure 13. Kinetic current normalizations. (**a**) Kinetic current density (**Supplementary Figure 11**) at 0.9 V (left) and 0.8 V (right) vs. RHE (average of cathodic and anodic scans) normalized by the AFM derived roughness factors (as deposited on glassy carbon). (**b**) Electrochemical double layer capacitance (EDLC) calculated at 0.65 V vs. RHE on the anodic CV sweep (black) and at 0.9 V vs. RHE (average of cathodic and anodic sweep) (green) as a function of Pd content. (**c**) Kinetic current at 0.9 V (left) and 0.8 V (right) vs. RHE (average of cathodic and anodic scans) normalized by the EDLC at 0.65 V vs. RHE on the anodic CV sweep, and (**d**) kinetic current at 0.9 V (left) and 0.8 V (right) vs. RHE (average of cathodic and anodic scans) normalized by the average EDLC at 0.9 V vs. RHE. (**e**) Pd-mass activity at 0.9 V vs. RHE relative to the total thin film Pd content (left y-axis) and relative to the estimated surface Pd coverage (right y-axis). (Relevant for **Figure 2c** in main text). All data points are representative of the average value from the cathodic and anodic scan of the third CV cycle and error bars are the standard deviation from measurements with separate samples (n = 2 samples for 0–30 at% Pd, and n = 3 for 40–100 at% Pd). (Relevant for **Figure 2c** in main text).

All observed trends in activity hold after various forms of normalization (**Supplementary Figure 13**). **Supplementary Figure 13b** shows that the EDLC is fairly constant within the technique limitations for the Ag-Pd system (on the same order of magnitude) for all thin films with different Ag-Pd composition.

Mass activity on **Supplementary Figure 13e** was estimated based on the Pd (density of 11.9 g cm^{-3}) volume content of the thin films with respect to their overall (left y-axis) or monolayer (right y-axis) thicknesses, 70 nm and ~ 0.3 nm respectively. Given that all thin films in this work were nominally flat and non-porous on the resolution of AFM, the surface-Pd mass activity (right y-axis, **Supplementary Figure 13e**) is likely a more representative activity metric than the total-Pd mass activity (left y-axis, **Supplementary Figure 13e**).

Supplementary Note 8: Stability/Durability Analysis

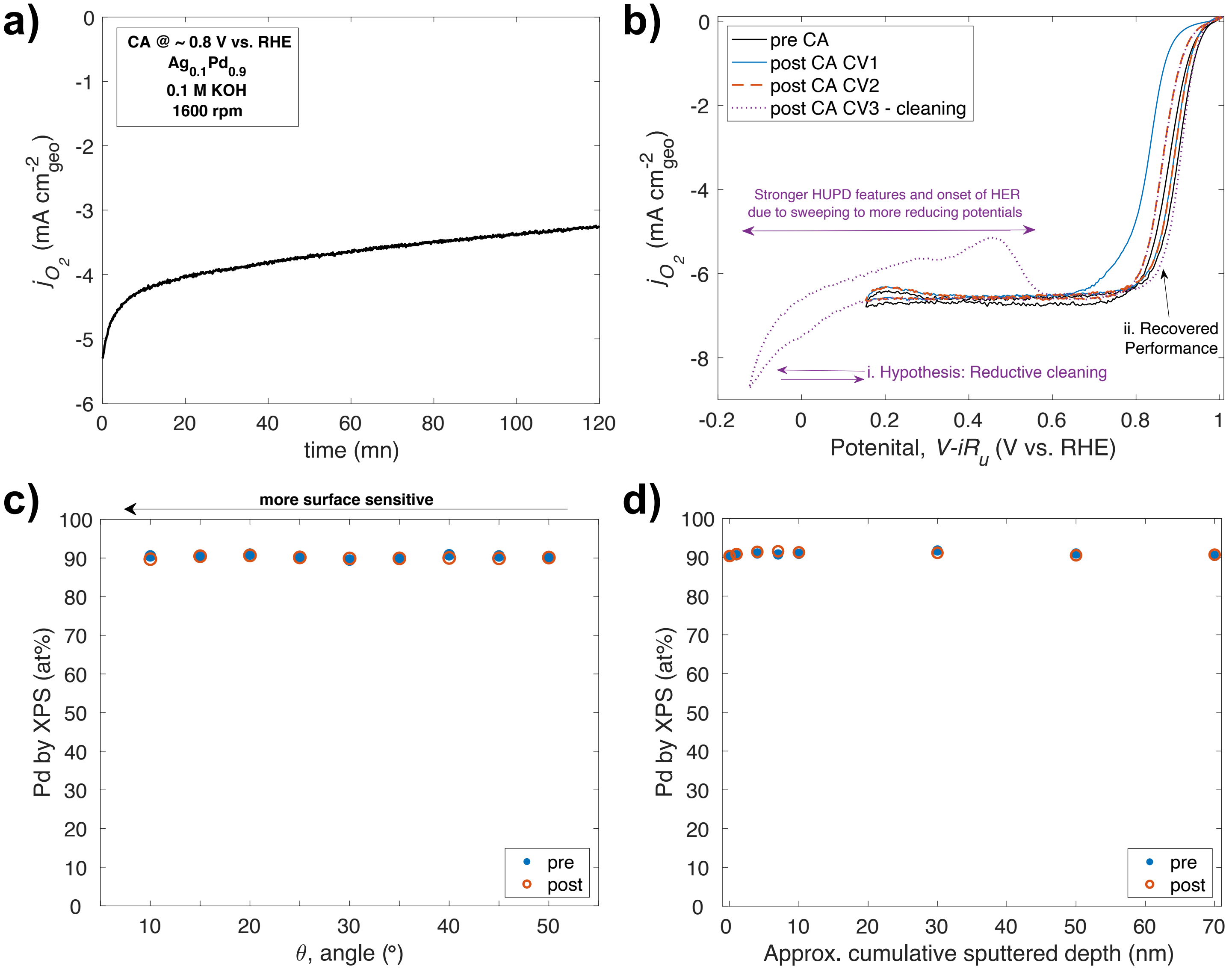

Supplementary Figure 14. Stability/durability examination of $Ag_{0.1}Pd_{0.9}$. (**a**) 2 hr chronoamperometry (CA) at ~0.8 V vs. RHE, (**b**) pre- and post-CA cyclic voltammetry (CV) tests. (**c**) Pre- (blue, filled) and post- (orange, unfilled) stability measurements (CA + CVs) angle resolved (AR) XPS Pd atomic composition as a function of sample-to-detector angle, θ. Note that $\theta = 45°$ is the standard/default collection angle for non-AR XPS measurements, with an estimated probe depth of 3.1 nm.[20] AR-XPS measurements were taken using a standard collection aperture because no peak signal was observed using the narrow collection aperture (slit cover on detector). The estimated probe depth at $\theta = 10°$ is ~ 0.8 nm[20]. We estimated probe (95% of electrons probed) depth as $d_{95\%} = 3\lambda\sin\theta$, based on the estimated $Ag_{0.1}Pd_{0.9}$ inelastic mean free path ($\lambda_{Ag_{0.1}Pd_{0.9}}$), and defined $\lambda_{Ag_{0.1}Pd_{0.9}} = 0.1\lambda_{Ag} + 0.9\lambda_{Pd} \approx 1.449$ nm, where $\lambda_{Ag} \approx 1.53$ nm and $\lambda_{Pd} \approx 1.44$ nm at an electron kinetic energy of 1096 eV (approximately equivalent to the binding energies corresponding to the Ag and Pd 3*d* peaks).[20] (**d**) Pd content (at%) as a function of cumulative sputtered depth pre- (blue, filled; separate sample from the same synthesis batch) and post- (orange, unfilled) stability measurements (CA + CVs) determined by XPS depth profiling (Ar^+ sputtering, sputtering rate calibrated versus SiO_2).

Representative Stability Testing of $Ag_{0.1}Pd_{0.9}$

For our best performing composition, $Ag_{0.1}Pd_{0.9}$, we performed chronoamperometry (CA) at ~ 0.8 V vs. RHE for 2 hrs (**Supplementary Figure 14a**) and saw a decrease in current of about 40% (interestingly, it is seen to decrease linearly after around 15 min). We hypothesize that the decrease in performance is due to reversible adventitious carbon uptake due to carbon corrosion from the graphite rod counter electrode, as well as possible Pd and/or Ag partial surface oxidation; the latter is in fact the main source of decreased performance in similarly high performing Pt-based ORR catalysts. ORR CVs pre- and post-CA testing (**Supplementary Figure 14b**) support this hypothesis, as 100% of the initial (pre-CA) performance was recovered by cycling the working electrode up to around -0.2 V vs. RHE to reductively clean off the adventitious carbon and any surface oxide on the film's surface. Additionally, AR-XPS (**Supplementary Figure 14c**) and XPS depth profiling (**Supplementary Figure 14d**) indicate that the composition of the film did not significantly change after stability testing. Altogether, our stability testing indicates that there is no intrinsic material or performance degradation in the timescale of or experiments.

Limited $Ag_{1-x}Pd_x$ Stability Testing

$Ag_{1-x}Pd_x$ thin film mechanical instabilities (not related to intrinsic activity) made our system not conducive for long-term durability testing. When cycling and chronoamperometric experiments were attempted the thin films partially or totally delaminated within three hours of testing. For all samples tested, we collected 3 ORR CVs as activity measurements. The first CV serves as a "cleaning" cycle, and the second and third serve as duplicate activity measurements for each sample tested (2 or 3 samples at each for all samples tested composition). These last 2 CVs were very reproducible for each sample and we report all our results using the third ORR CV cycle for each sample.

To assess the material response to electrochemical testing, we characterized the structural, morphological, and compositional surface changes after electrocatalysis (3 CVs in O_2-saturated 0.1 M KOH, followed by 2 CVs in N_2-saturated electrolyte, followed by 5 CVs at different scan rates in N_2-saturated electrolyte, all at 1600 rpm) via XPS, GI-XRD, and AFM, respectively. Respectively, XPS (**Supplementary Figure 4**), GI-XRD (**Supplementary Figure 1**), and AFM (**Supplementary Figure 6** and **Supplementary Table 2**) suggest that the composition, structure and extent of alloying, and surface smoothness of our thin film $Ag_{1-x}Pd_x$ electrocatalysts remain largely unchanged (within instrument sensitivity) after undergoing ORR activity characterization experiments. This post-characterization suggests that our catalyst surface does not change on the timescale of our electrochemical measurements and indicates that the ex-situ material properties can be meaningfully related to the activity trends.

Supplementary Note 9: Comparison to State-of-the-Art Specific Activity

Supplementary Table 4. Comparison to a selection of high-performing ORR catalysts in 0.1 M KOH (or similar) using a RDE setup. We only report representative catalysts among the most active found in the literature, where kinetic current density is given or easily calculated and explicitly normalized by a physically representative exposed catalyst (or active site) surface area ("*cat*") (also referred to as electrochemically active surface area (ECSA)). Examples of ECSA techniques are AFM surface roughness for thin films and flat surfaces, electrochemical stripping (e.g of CO or Cu), hydrogen under-potential deposition, metal-oxide layer reduction, and scan rate potential-cycling.[21,22] In the absence of AFM, geometric electrode area was taken as an acceptable approximate ECSA only for well-defined single crystal catalysts. Outside of this work, references are for linear sweep voltammetry data. For our Ag-Pd thin films in this table, we report the average (from the cathodic and anodic cycle) specific activity; see **Supplementary Table 3** for the individual activity at each cycle leg (our anodic/forward cycle shows slightly higher activities than the average we report in this table). Nomenclature: (111) = (111) oriented single crystal, @ = on/at/on top, NPs = nanoparticles, NCs = nanocrystals, / = supported on (e.g. Pt/C = Pt supported on carbon).

Catalyst	Specific Activity (mA cm_{cat}^{-2}) @0.9 V vs. RHE	Electrolyte	Scan Rate (mV s^{-1})	Reference
**PdMo bimetallane/C**	11.7	0.1 M KOH	20	23
**$Ag_{0.1}Pd_{0.9}$**	3.46	0.1 M KOH	20	this work
**Pt(111)**	3.23	0.1 M KOH	50	24
**Pd_3Bi**	2.3	0.1 M KOH	20	25
**PdCu/C**	2.30	1 M KOH	5	16
**$Ag_{0.2}Pd_{0.8}$**	1.96	0.1 M KOH	20	this work
**(activated) $PtCu_3$ NPs/C**	1.95	0.1 M KOH	5	26
**$Ag_{0.3}Pd_{0.7}$**	1.24	0.1 M KOH	20	this work
**$Ag_{0.4}Pd_{0.6}$**	1.18	0.1 M KOH	20	this work
**Pd/C**	0.92	1 M KOH	5	16
**Pd**	0.77	0.1 M KOH	20	this work
**$Ag_{0.5}Pd_{0.5}$**	0.67	0.1 M KOH	20	this work
**cubic Pd NPs**	0.56	0.1 M KOH	10	27
**Pd/C**	0.52	0.1 M KOH	20	25
**Pt_3Cu NPs/C**	0.50	0.1 M KOH	5	26
**Pd metallane/C**	0.48	0.1 M KOH	20	23
**$Pd/W_{18}O_{49}$**	0.45	0.1 M KOH	10	28
**Bulk Pd**	0.42	0.1 M KOH	10	27
**PdPt Alloy NCs**	0.40	0.1 M KOH	10	29
**Pd/C-600**	0.39	0.1 M NaOH	10	30

**PtCu NPs/C**	0.33	0.1 M KOH	5	26
**Pt NPs/C**	0.33	0.1 M KOH	5	26
**Pt(111)**	0.28	0.1 M NaOH	5	31
**Commercial Pt/C**	0.27	0.1 M KOH	20	23
**60 wt% Pd/GNS**	0.25	0.1 M NaOH	10	32
**Pd/C-500**	0.23	0.1 M NaOH	10	30
**20% Pt/C**	0.21	0.1 M KOH	10	29
**$Ag_{0.6}Pd_{0.4}$**	0.20	0.1 M KOH	20	this work
**Pd/C-400**	0.20	0.1 M NaOH	10	30
**spherical Pd NPs**	0.20	0.1 M KOH	10	27
**40 wt% Pt/C**	0.19	0.1 M KOH	10	33
**Pd@PdPt NCs**	0.19	0.1 M KOH	10	29
**Pt/C**	0.18	0.1 M KOH	20	34
**Pd/C-300**	0.17	0.1 M NaOH	10	30
**60 wt% Pt/GNS**	0.16	0.1 M NaOH	10	32
**Pd/C-AR**	0.14	0.1 M NaOH	10	30
**Commercial Pd/C**	0.14	0.1 M KOH	20	23
**Ni@Pd_3/C NPs**	0.13	0.1 M KOH	20	34
**$PtCu_3$ NPs/C**	0.13	0.1 M KOH	5	26
**Pt/C**	0.12	0.1 M KOH	20	25
**Bulk Pt disk**	0.12	0.1 M KOH	10	33
**Pt/TiO_2-FGS**	0.11	0.1 M KOH	10	33
**Ni@Pd_2 NPs/C**	0.11	0.1 M KOH	20	34
**Ni_3@Pd NPs/C**	0.10	0.1 M KOH	20	34
**Ni@Pd NPs/C**	0.08	0.1 M KOH	20	34
**$Ag_{0.7}Pd_{0.3}$**	0.07	0.1 M KOH	20	this work
**Pd NPs/C**	0.06	0.1 M KOH	20	34
**CoZn-NC 700**	0.05	0.1 M KOH	5	35

Supplementary Note 10: Density Functional Theory Modeling

To evaluate if the Ag-rich surfaces investigated in this study are susceptible to surface segregation, the segregation energies for a bulk Pd atom in a Ag(111) lattice were calculated at vacuum and in the presence of an adsorbed *OH. Segregation of a bulk Pd atom to the surface of Ag(111) was found to be unfavorable under vacuum conditions, while, in the presence of adsorbed *OH, segregation is slightly thermodynamically favorable. This indicates that the surface layer generally prefers to maximize the available Ag content under vacuum conditions, while Pd sites are stabilized on the surface in the presence of adsorbed *OH. In contrast, XPS (**Supplementary Figures 4**, **14c**, and **14d**) and GI-XRD (**Figure 1b** and **Supplementary Figure 1c**) data suggest that, within technique sensitivity (top several nanometers), the Ag-Pd alloy thin films did not undergo major surface rearrangements during ORR experiments.

Interestingly, the limiting potential for the ORR on Pd_{atom}−Ag (−5%) and Pd_{layer}−Ag(111) were similar to those on Pt(111) and Pd(111). These two Ag-Pd configurations (Pd_{atom}−Ag (−5%) and Pd_{layer}−Ag) could serve as a strategy to further reduce Pd loading in Ag-Pd bimetallic systems without large activity losses. Specifically, this motivates studying monodispersed Pd on a Ag matrix grown under lattice compression, and further investigating systems such as Pd@Ag core-shell nanoparticles (or layered thin films).

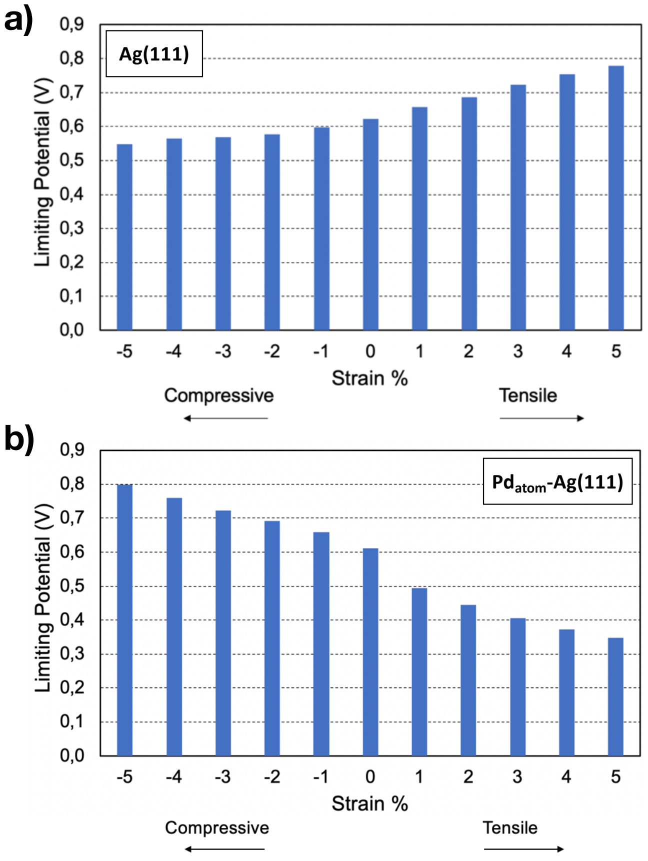

Supplementary Figure 15. Select strain effect calculations. (**a**) Effect of compressive and tensile strain on the calculated limiting potentials for Ag(111) surface. (**b**) Effect of compressive and tensile strain on the calculated limiting potentials for Pd_{atom}-Ag(111) surface. (Relevant for **Figure 3** in main text).

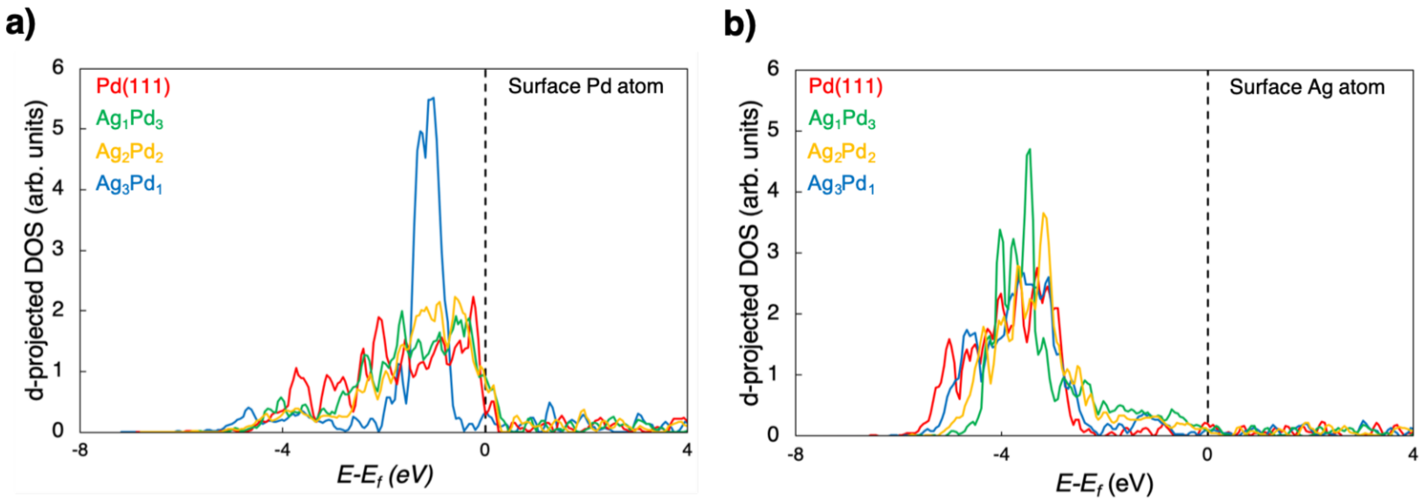

Supplementary Figure 16. Ag-Pd d-projected density of states. d-projected density of states (PDOS) for (**a**) Pd atom in Pd(111), Ag_1Pd_3(111), Ag_2Pd_2(111), and Ag_3Pd_1(111), and (**b**) Ag atom in Ag(111), Ag_1Pd_3(111), Ag_2Pd_2(111), and Ag_3Pd_1(111). (Relevant for **Figure 3** in main text).

Supplementary Figure 16a indicates that the d-projected density of states of Pd atom in Pd(111), Ag_1Pd_3(111), and Ag_2Pd_2(111) are similar while Ag_3Pd_1(111) is significantly different. The d-projected density of states of Pd atom in Ag_3Pd_1(111) resembles the sharp peak observed on Pd atom in Pd_{atom}-Ag(111) surface (Figure S10.3) though a slight broadening and decrease in intensity is observed. This broadening could be attributed to the compensating effects of compressive strain in Ag_3Pd_1(111) compared to Pd_{atom}-Ag(111). A slightly lower *d*-band edge relative to the Fermi level is observed for Ag_1Pd_3(111) and Ag_2Pd_2(111) indicating a weaker oxygen adsorption on Pd atom which resulted in increase in activity (**Figure 3b**). On the other hand, **Figure 1b** shows rather similar d-projected density of states of Ag atom in Ag(111) and Ag_3Pd_1(111) surfaces while the Ag atom in Ag_1Pd_3(111) and Ag_2Pd_2(111) shows a slight narrowing of the *d*-band and slightly lower *d*-band edge relative to the Fermi level.

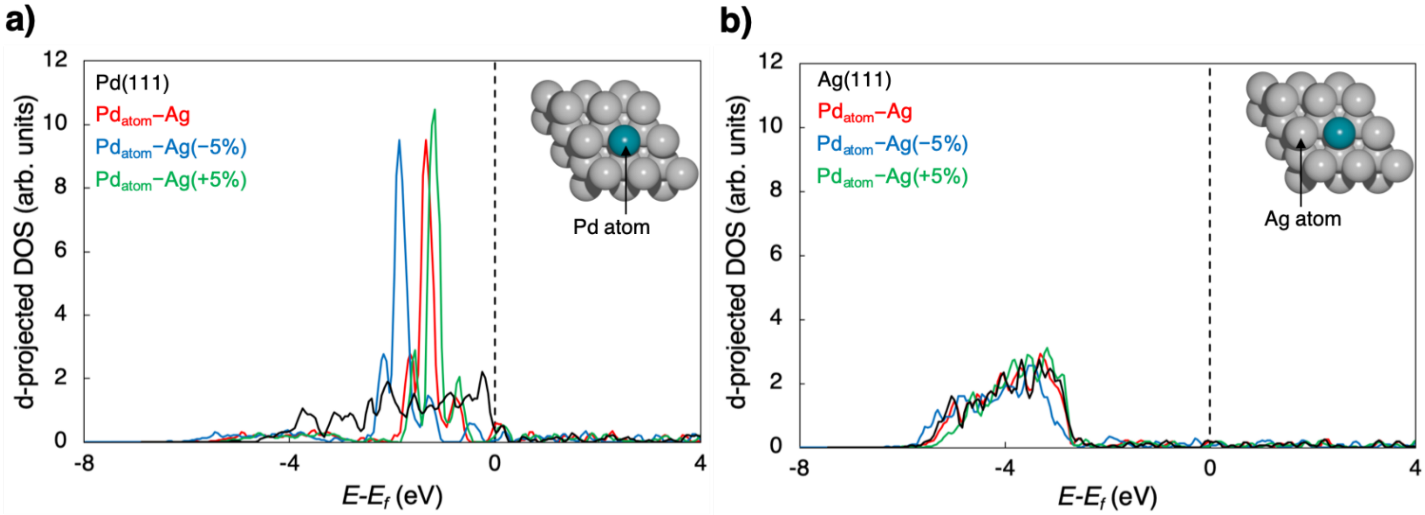

Supplementary Figure 17. Pd_{atom}-Ag(111) d-projected density of states. d-projected density of states (PDOS) for (**a**) Pd atom in Pd(111) and Pd_{atom}-Ag(111) surface with 5%, 0%, -5% strain. (**b**) Ag atom in Ag(111) and Pd_{atom}-Ag(111) surface with 5%, 0%, -5% strain. (Relevant for **Figure 3** in main text).

Supplementary Figure 17a indicates that the d-projected density of states of Pd atom in Pd_{atom}-Ag(111) surface is significantly different from a Pd atom in Pd(111) surface and a formation of a sharp peak near the Fermi level.

This peak could result from the ineffective mixing of the electron densities of the Pd single atom and the Ag(111) surface and the effective tensile strain on the Pd single atom due to large lattice of Ag(111). A lower *d*-band edge relative to the Fermi level is observed for Pd single atom in -5% compressively strained Ag(111), Pd_{atom}-Ag(-5%), compared to Pd_{atom}-Ag and Pd_{atom}-Ag(5%) indicating a weaker oxygen adsorption on Pd atom. This results in increase in ORR overpotential and therefore increased activity. A similar d-projected density of states of Ag atom in Ag(111) and Pd_{atom}-Ag(111) surfaces with different strains is observed (**Supplementary Figure 17b**) indicating that the ORR activity of these Ag atoms are not affected.

Supplementary Table 5. DFT adsorption free energies of OH* and OOH*, and thermodynamic limiting potentials and steps for considered Ag−Pd active site models in **Figure 3a**. Note that all the considered surfaces are (111) facets. (This is the data plotted on **Figure 3b** in the main text).

Active site model	ΔG_{OH*} (eV)	ΔG_{OOH*} (eV)	U_L (V)	Potential limiting step
**Pt**[36]	0.80	4.00	0.80	$OH^* + (H^+ + e^-) \rightarrow H_2O + *$
**Pd**	0.79	4.12	0.79	$OH^* + (H^+ + e^-) \rightarrow H_2O + *$
**Ag**	1.10	4.31	0.61	$O_2 + (H^+ + e^-) + * \rightarrow OOH^*$
**Ag(+5%)**	0.94	4.19	0.73	$O_2 + (H^+ + e^-) + * \rightarrow OOH^*$
**Ag(−5%)**	1.17	4.45	0.47	$O_2 + (H^+ + e^-) + * \rightarrow OOH^*$
**Pd_{atom}−Ag**	0.63	3.99	0.63	$OH^* + (H^+ + e^-) \rightarrow H_2O + *$
**Pd_{atom}−Ag(+5%)**	0.37	3.65	0.37	$OH^* + (H^+ + e^-) \rightarrow H_2O + *$
**Pd_{atom}−Ag(−3%)**	0.72	4.04	0.72	$OH^* + (H^+ + e^-) \rightarrow H_2O + *$
**Pd_{atom}−Ag(−5%)**	0.80	4.12	0.80	$OH^* + (H^+ + e^-) \rightarrow H_2O + *$
**Pd_{layer}−Ag**	0.83	4.14	0.78	$O_2 + (H^+ + e^-) + * \rightarrow OOH^*$
**Ag_{layer}−Pd**	1.19	4.47	0.45	$O_2 + (H^+ + e^-) + * \rightarrow OOH^*$
**Ag_3Pd_1**	0.72	3.99	0.72	$OH^* + (H^+ + e^-) \rightarrow H_2O + *$
**Ag_2Pd_2**	0.82	4.09	0.82	$OH^* + (H^+ + e^-) \rightarrow H_2O + *$
**Ag_1Pd_3**	0.83	4.07	0.83	$OH^* + (H^+ + e^-) \rightarrow H_2O + *$

Supplementary Table 6. Effective Bader charges (q) defined as $q = Z_{val} - q_{Bader}$, where Z_{val} is the number of valence electrons determined by POTCAR file (pseudopotential for each atomic species) in VASP and q_{Bader} is the computed Bader charge for few considered Ag−Pd active site models in **Figure 3a**. Note that all the considered surfaces are (111) facets.

Active site model	Effective Bader charge (e)	
	Ag atom	Pd atom
**Pd**	–	−0.02
**Ag**	−0.02	–
**Ag(+5%)**	−0.03	–
**Ag(−5%)**	−0.01	–
**Pd_{atom}−Ag**	0.00	−0.23
**Ag_3Pd_1**	0.04	−0.22
**Ag_2Pd_2**	0.10	−0.15
**Ag_1Pd_3**	0.13	−0.08

DFT Methodology Notes. All the DFT calculations performed in this study are spin polarized. Specifically, we use the ISPIN = 2 tag to turn on the spin and then define the magnetic moment using the MAGMOM tag as implemented in VASP. Because no drastic change in observed activity trends is expected due to valence spin orbits, in this study we do not consider valence spin orbits.[37] We do not account for dispersion interactions because they are expected to only have a minor effect and generally calculations for chemisorbed species using RPBE and BEEF–vdW (which accounts for dispersion interactions) follow similar trends.[38] While adsorbate-adsorbate interactions could play a role in the apparent catalyst activity, we anticipate similar adsorbate−adsorbate interactions across all the metal surfaces considered in this study due to the similarity in surface atoms ordering (all considered surfaces are (111) facets) and hence similarity in the resulting adsorbate−adsorbate distances. Therefore, adsorbate−adsorbate interactions would likely not change the calculated activity trends. The aim of this work is to look at activity trends across the Ag-Pd compositional spectrum and therefore a detailed analysis considering adsorbate−adsorbate interactions for all the individual surfaces is beyond the scope of this study.

Supplementary Information References

1. Evans, D. M. & Wilman, H. Crystal growth and orientation in deposits condensed from the vapour. *Acta Crystallogr.* **5**, 731–738 (1952).
2. Thornton, J. A. Influence of apparatus geometry and deposition conditions on the structure and topography of thick sputtered coatings. *J. Vac. Sci. Technol.* **11**, 666–670 (1974).
3. Morgan, D., Mayeshiba, T. & Morgan, D. DFT dilute solute diffusion in Al, Cu, Ni, Pd, Pt, Mg, Fe, W, Mo, Au, Ca, Ir, Pb, Ag, Zr. figureshare.com, https://doi.org/10.6084/m9.figshare.1546772.v10 (2018).
4. Wu, H., Mayeshiba, T. & Morgan, D. High-Throughput ab-initio dilute solute diffusion database. *Sci. Data* **3**, 1–11 (2016).
5. Stöhr, J. *NEXAFS SPECTROSCOPY*. (Springer-Verlag, Berlin/Heidelberg, 1992).
6. Witjens, L. C., Bitter, J. H., Van Dillen, A. J., De Jong, K. P. & De Groot, F. M. F. Pd L3 edge XANES investigation of the electronic and geometric structure of Pd/Ag-H membranes. *Phys. Chem. Chem. Phys.* **6**, 3903–3906 (2004).
7. Coulthard, I. & Sham, T. K. Charge Redistribution in Pd-Ag Alloys from a Local Perspective. *Phys. Rev. Lett.* **77**, 4824–4827 (1996).
8. Zhou, R., Zheng, Y., Jaroniec, M. & Qiao, S. Z. Determination of the Electron Transfer Number for the Oxygen Reduction Reaction: From Theory to Experiment. *ACS Catal.* **6**, 4720–4728 (2016).
9. Zhang, J. *PEM FUEL CELL ELECTROCATALYSTS AND CATALYST LAYERS: FUNDAMENTALS AND APPLICATIONS*. (Springer-Verlag, London, 2008).
10. Jovic, B. M., Jovic, V. D. & Stafford, G. R. Cyclic voltammetry on Ag(111) and Ag(100) faces in sodium hydroxide solutions. *Electrochem. Commun.* **1**, 247–251 (1999).
11. Horswell, S. L., Pinheiro, A. L. N., Savinova, E. R., Danckwerts, M., Pettinger, B., Zei, M.-S. & Ertl, G. A Comparative Study of Hydroxide Adsorption on the (111), (110), and (100) Faces of Silver with Cyclic Voltammetry, Ex Situ Electron Diffraction, and In Situ Second Harmonic Generation. *Langmuir* **20**,

10970–10981 (2004).

12. Higgins, D., Wette, M., Gibbons, B. M., Siahrostami, S., Hahn, C., Escudero-Escribano, M., García-Melchor, M., Ulissi, Z., Davis, R. C., Mehta, A., Clemens, B. M., Nørskov, J. K. & Jaramillo, T. F. Copper Silver Thin Films with Metastable Miscibility for Oxygen Reduction Electrocatalysis in Alkaline Electrolytes. *ACS Appl. Energy Mater.* **1**, 1990–1999 (2018).

13. Savinova, E. R., Scheybal, A., Danckwerts, M., Wild, U., Pettinger, B., Doblhofer, K., Schlögl, R. & Ertl, G. Structure and dynamics of the interface between a Ag single crystal electrode and an aqueous electrolyte. *Faraday Discuss.* **121**, 181–198 (2002).

14. Grdeń, M., Łukaszewski, M., Jerkiewicz, G. & Czerwiński, A. Electrochemical behaviour of palladium electrode: Oxidation, electrodissolution and ionic adsorption. *Electrochim. Acta* **53**, 7583–7598 (2008).

15. Diculescu, V. C., Chiorcea-Paquim, A. M., Corduneanu, O. & Oliveira-Brett, A. M. Palladium nanoparticles and nanowires deposited electrochemically: AFM and electrochemical characterization. *J. Solid State Electrochem.* **11**, 887–898 (2007).

16. Yang, Y., Chen, G., Zeng, R., Villarino, A. M., DiSalvo, F. J., van Dover, R. B. & Abruña, H. D. Combinatorial Studies of Palladium-Based Oxygen Reduction Electrocatalysts for Alkaline Fuel Cells. *J. Am. Chem. Soc.* **142**, 3980–3988 (2020).

17. Betancourt, L. E., Rojas-Pérez, A., Orozco, I., Frenkel, A. I., Li, Y., Sasaki, K., Senanayake, S. D. & Cabrera, C. R. Enhancing ORR performance of bimetallic PdAg electrocatalysts by designing interactions between Pd and Ag. *ACS Appl. Energy Mater.* **3**, 2342–2349 (2020).

18. Xing, X.-L., Zhao, Y.-F., Li, H., Wang, C.-T., Li, Q.-X. & Cai, W.-B. High Performance Ag Rich Pd-Ag Bimetallic Electrocatalyst for Ethylene Glycol Oxidation in Alkaline Media. *J. Electrochem. Soc.* **165**, J3259–J3265 (2018).

19. Bard, A. J. & Faulkner, L. R. *ELECTOCHEMICAL METHODS: FUNDAMENTLAS AND APPLICATIONS*. (Wiley, New York, 2000).

20. Shinotsuka, H., Tanuma, S., Powell, C. J. & Penn, D. R. Calculations of electron inelastic mean free paths. X. Data for 41 elemental solids over the 50 eV to 200 keV range with the relativistic full Penn algorithm. *Surf. Interface Anal.* **47**, 871–888 (2015).

21. Łukaszewski, M., Soszko, M. & Czerwiński, A. Electrochemical Methods of Real Surface Area Determination of Noble Metal Electrodes-an Overview. *Int. J. Electrochem. Sci* **11**, 4442–4469 (2016).

22. Yoon, Y., Yan, B. & Surendranath, Y. Suppressing Ion Transfer Enables Versatile Measurements of Electrochemical Surface Area for Intrinsic Activity Comparisons. *J. Am. Chem. Soc.* **140**, 2397–2400 (2018).

23. Luo, M., Zhao, Z., Zhang, Y., Sun, Y., Xing, Y., Lv, F., Yang, Y., Zhang, X., Hwang, S., Qin, Y., Ma, J. Y., Lin, F., Su, D., Lu, G. & Guo, S. PdMo bimetallene for oxygen reduction catalysis. *Nature* **574**, 81–85 (2019).

24. Marković, N. M., Schmidt, T. J., Stamenković, V. & Ross, P. N. Oxygen Reduction Reaction on Pt and Pt Bimetallic Surfaces: A Selective Review. *Fuel Cells* **1**, 105–116 (2001).

25. Sun, D., Wang, Y., Livi, K. J. T., Wang, C., Luo, R., Zhang, Z., Alghamdi, H., Li, C., An, F., Gaskey, B., Mueller, T. & Hall, A. S. Ordered Intermetallic Pd3Bi Prepared by an Electrochemically Induced Phase Transformation for Oxygen Reduction Electrocatalysis. *ACS Nano* **13**, 10818–10825 (2019).

26. Oezaslan, M., Hasché, F. & Strasser, P. PtCu3 , PtCu and Pt3Cu Alloy Nanoparticle Electrocatalysts for Oxygen Reduction Reaction in Alkaline and Acidic Media. *J. Electrochem. Soc.* **159**, B444–B454 (2012).

27. Erikson, H., Sarapuu, A., Alexeyeva, N., Tammeveski, K., Solla-Gullón, J. & Feliu, J. M. Electrochemical reduction of oxygen on palladium nanocubes in acid and alkaline solutions. *Electrochim. Acta* **59**, 329–335 (2012).

28. Lu, Y., Jiang, Y., Gao, X., Wang, X. & Chen, W. Strongly coupled pd nanotetrahedron/tungsten oxide nanosheet hybrids with enhanced catalytic activity and stability as oxygen reduction electrocatalysts. *J. Am. Chem. Soc.* **136**, 11687–11697 (2014).

29. Liu, Y., Liu, S., Che, Z., Zhao, S., Sheng, X., Han, M. & Bao, J. Concave octahedral Pd@PdPt electrocatalysts integrating core-shell, alloy and concave structures for high-efficiency oxygen reduction and hydrogen evolution reactions. *J. Mater. Chem. A* **4**, 16690–16697 (2016).
30. Jiang, L., Hsu, A., Chu, D. & Chen, R. Size-Dependent Activity of Palladium Nanoparticles for Oxygen Electroreduction in Alkaline Solutions. *J. Electrochem. Soc.* **156**, B643 (2009).
31. Lima, F. H. B., Zhang, J., Shao, M. H., Sasaki, K., Vukmirovic, M. B., Ticianelli, E. A. & Adzic, R. R. Catalytic activity - d-band center correlation for the O2 reduction reaction on platinum in alkaline solutions. *J. Phys. Chem. C* **111**, 404–410 (2007).
32. Seo, M. H., Choi, S. M., Kim, H. J. & Kim, W. B. The graphene-supported Pd and Pt catalysts for highly active oxygen reduction reaction in an alkaline condition. *Electrochem. Commun.* **13**, 182–185 (2011).
33. Tiido, K., Alexeyeva, N., Couillard, M., Bock, C., MacDougall, B. R. & Tammeveski, K. Graphene-TiO2 composite supported Pt electrocatalyst for oxygen reduction reaction. *Electrochim. Acta* **107**, 509–517 (2013).
34. Jiang, J., Gao, H., Lu, S., Zhang, X., Wang, C. Y., Wang, W. K. & Yu, H. Q. Ni-Pd core-shell nanoparticles with Pt-like oxygen reduction electrocatalytic performance in both acidic and alkaline electrolytes. *J. Mater. Chem. A* **5**, 9233–9240 (2017).
35. Chen, B., He, X., Yin, F., Wang, H., Liu, D.-J., Shi, R., Chen, J. & Yin, H. MO-Co@N-Doped Carbon (M = Zn or Co): Vital Roles of Inactive Zn and Highly Efficient Activity toward Oxygen Reduction/Evolution Reactions for Rechargeable Zn-Air Battery. *Adv. Funct. Mater.* **27**, 1700795 (2017).
36. Kulkarni, A., Siahrostami, S., Patel, A. & Nørskov, J. K. Understanding Catalytic Activity Trends in the Oxygen Reduction Reaction. *Chem. Rev.* **118**, 2302–2312 (2018).
37. Philipsen, P., van Lenthe, E., Snijders, J. & Baerends, E. Relativistic calculations on the adsorption of CO on the (111) surfaces of Ni, Pd, and Pt within the zeroth-order regular approximation. *Phys. Rev. B - Condens. Matter Mater. Phys.* **56**, 13556–13562 (1997).

38. Wellendorff, J., Lundgaard, K. T., Jacobsen, K. W. & Bligaard, T. MBEEF: An accurate semi-local Bayesian error estimation density functional. *J. Chem. Phys.* **140**, 144107 (2014).
